# Supplementary material for: Non‐Hermitian Global Synchronization
Source: Adv Sci (Weinh). 2024 Nov 16;12(2):2408460. doi: 10.1002/advs.202408460 (PMC11727122; doi:10.1002/advs.202408460)
Supplement: Supplementary file 1 — Supporting Information [file ADVS-12-2408460-s001.docx]

**Supporting Information****: Non-Hermitian global synchronization**

Weixuan Zhang, Fengxiao Di, and Xiangdong Zhang*

Supporting Information 1. Linear stability analysis of non-Hermitian skin synchronized states.

Supporting Information 2. Numerical results of the order parameter *R*(*t*) as a function of the lattice length.

Supporting Information 3. Numerical results of nonlinear dynamics with weak and strong Hatano-Nelson-typed couplings.

Supporting Information 4. Numerical results of nonlinear dynamics with Hatano-Nelson-typed couplings at different lattice lengths.

Supporting Information 5. Nonlinear eigenstates of the model with Hatano-Nelson-typed couplings based on Newton-gradient method.

Supporting Information 6. The relationship between IPRs of non-Hermitian SSH model and order-parameters.

Supporting Information 7. Nonlinear eigenstates of the model with non-Hermitian SSH-typed couplings based on Newton-gradient method.

Supporting Information 8. The robustness of non-Hermitian topological global ﻿synchronization.

Supporting Information 9. Numerical results of nonlinear dynamics with non-Hermitian SSH-typed couplings at different lattice lengths.

Supporting Information 10. The correspondence between Chua diode and Stuart-Landau oscillator.

Supporting Information 11. The derivation of eigenequation for the non-Hermitian ﻿synchronized circuit.

Supporting Information 12. Simulation results of non-Hermitian skin ﻿synchronization in electric circuits.

Supporting Information 13. Simulation results of non-Hermitian topological global ﻿synchronization in electric circuits.

Supporting Information 14. Non-Hermitian skin ﻿synchronization in one-dimensional lattices with onsite loss and gain.

Supporting Information 15. Sample fabrications and circuit measurements.

**Supporting Information 1. Linear stability analysis of non-Hermitian skin synchronized states.** In this part, we provide the detailed procedure for the linear stability analysis of non-Hermitian synchronized states. Eq. (1) can be written in $\dot{\boldsymbol{Z}}=A(\boldsymbol{Z})$ with $\boldsymbol{Z}$ being the state vector composed of real ($Z_{l}^{r}$) and imaginary ($Z_{l}^{i}$) parts of all $Z_{l}$ as $\boldsymbol{Z}\mathbf{=[}Z_{1}^{r}\mathbf{,}Z_{1}^{i}\boldsymbol{,\ldots,}Z_{N}^{r}\mathbf{,}Z_{N}^{i}\mathbf{]}$ and $A(\boldsymbol{Z})$ being a set of 2*N* state equations:

$A\left( \boldsymbol{Z} \right)=\left[ \begin{matrix} \left( i\omega+\alpha\right)Z_{1}^{r}-\beta\left( {Z_{1}^{r}}^{3}+Z_{1}^{r}{Z_{1}^{i}}^{2} \right)+J_{+}Z_{2}^{i} \\ \left( i\omega+\alpha\right)Z_{1}^{i}-\beta\left( {Z_{1}^{r}}^{2}Z_{1}^{i}+{Z_{1}^{i}}^{3} \right)-J_{+}Z_{2}^{r} \\ \left( i\omega+\alpha\right)Z_{2}^{r}-\beta\left( {Z_{2}^{r}}^{3}+Z_{2}^{r}{Z_{2}^{i}}^{2} \right)+J_{+}Z_{3}^{i}+J_{-}Z_{1}^{i} \\ \left( i\omega+\alpha\right)Z_{2}^{i}-\beta\left( {Z_{2}^{r}}^{2}Z_{2}^{i}+{Z_{2}^{i}}^{3} \right)+\left( -J_{+}Z_{3}^{r}-J_{-}Z_{1}^{r} \right) \\ \vdots\\ \vdots\\ \left( i\omega+\alpha\right)Z_{N}^{r}-\beta\left( {Z_{N}^{r}}^{3}+Z_{N}^{r}{Z_{N}^{i}}^{2} \right)+J_{-}Z_{N-1}^{i} \\ \left( i\omega+\alpha\right)Z_{N}^{i}-\beta\left( {Z_{N}^{r}}^{2}Z_{N}^{i}+{Z_{N}^{i}}^{3} \right)-J_{-}Z_{N-1}^{r} \end{matrix} \right]_{2N\times1}$ (1)

We further linearize the equation $\dot{\boldsymbol{Z}}=A(\boldsymbol{Z})$ around the anti-phase ($\boldsymbol{Z}_{\boldsymbol{an}}\boldsymbol{=[}Z_{an,1}^{r}\mathbf{,}Z_{an,1}^{i}\boldsymbol{,\ldots,}Z_{an,N}^{r}\mathbf{,}Z_{an,N}^{i}\boldsymbol{]}$) and in-phase ($\boldsymbol{Z}_{\boldsymbol{in}}\boldsymbol{=[}Z_{in,1}^{r}\mathbf{,}Z_{in,1}^{i}\boldsymbol{,\ldots,}Z_{in,N}^{r}\mathbf{,}Z_{in,N}^{i}\boldsymbol{]}$) synchronized states, where the corresponding spatial distributions of real and imaginary parts of $\boldsymbol{Z}_{\boldsymbol{an}}$ and $\boldsymbol{Z}_{\boldsymbol{in}}$ are presented in Figures S1a and S1b, respectively. In this case, the linearized state equations around $\boldsymbol{Z}_{\boldsymbol{an}}$ and $\boldsymbol{Z}_{\boldsymbol{in}}$ become $\dot{\boldsymbol{Z}_{\boldsymbol{\delta}}}=\boldsymbol{J(}\boldsymbol{Z}_{\boldsymbol{an}}\boldsymbol{)}\boldsymbol{Z}_{\boldsymbol{\delta}}$ and $\dot{\boldsymbol{Z}_{\boldsymbol{\delta}}}=\boldsymbol{J(}\boldsymbol{Z}_{\boldsymbol{in}}\boldsymbol{)}\boldsymbol{Z}_{\boldsymbol{\delta}}$ with $\boldsymbol{Z}_{\boldsymbol{\delta}}\boldsymbol{=Z-}\boldsymbol{Z}_{\boldsymbol{an}}$ and $\boldsymbol{Z}_{\boldsymbol{\delta}}\boldsymbol{=Z-}\boldsymbol{Z}_{\boldsymbol{in}}$ . In addition, the Jacobian matrixes $\boldsymbol{J}\left( \boldsymbol{Z}_{\boldsymbol{an}} \right)$and $\boldsymbol{J}(\boldsymbol{Z}_{\boldsymbol{in}})$ of anti-phase and in-phase synchronized states are expressed as

$$\boldsymbol{J}\left( \boldsymbol{Z}_{\boldsymbol{an}} \right)=\left( i\omega+\alpha\right)\boldsymbol{I}_{\boldsymbol{2}\boldsymbol{N\times2}\boldsymbol{N}}\boldsymbol{+}$$

$$\left[ \begin{matrix} -3\beta{Z_{an,1}^{r}}^{2}-\beta{Z_{an,1}^{i}}^{2} & -2\beta Z_{an,1}^{r}Z_{an,1}^{i} & 0 & J_{+} & \ddots& \ddots& 0 & 0 \\ -2\beta Z_{an,1}^{r}Z_{an,1}^{i} & -3\beta{Z_{an,1}^{i}}^{2}-\beta{Z_{an,1}^{r}}^{2} & -J_{+} & 0 & \ddots& \ddots& 0 & 0 \\ 0 & J_{-} & -3\beta{Z_{an,2}^{r}}^{2}-\beta{Z_{an,2}^{i}}^{2} & -2\beta Z_{an,2}^{r}Z_{an,2}^{i} & \ddots& \ddots& 0 & 0 \\ -J_{-} & 0 & -2\beta Z_{an,2}^{r}Z_{an,2}^{i} & -3\beta{Z_{an,2}^{i}}^{2}-\beta{Z_{an,2}^{r}}^{2} & \ddots& \ddots& 0 & 0 \\ \ddots& \ddots& \ddots& \ddots& \ddots& \ddots& 0 & J_{+} \\ \ddots& \ddots& \ddots& \ddots& \ddots& \ddots& -J_{+} & 0 \\ 0 & 0 & 0 & 0 & 0 & J_{-} & -3\beta{Z_{an,N}^{r}}^{2}-\beta{Z_{an,N}^{i}}^{2} & -2\beta Z_{an,N}^{r}Z_{an,N}^{i} \\ 0 & 0 & 0 & 0 & -J_{-} & 0 & -2\beta Z_{an,N}^{r}Z_{an,N}^{i} & -3\beta{Z_{an,N}^{i}}^{2}-\beta{Z_{an,N}^{r}}^{2} \end{matrix} \right]_{2N\times2N}$$

$\boldsymbol{J}\left( \boldsymbol{Z}_{\boldsymbol{in}} \right)=\left( i\omega+\alpha\right)\boldsymbol{I}_{\boldsymbol{2}\boldsymbol{N\times2}\boldsymbol{N}}\boldsymbol{+}$

$\left[ \begin{matrix} -3\beta{Z_{in,1}^{r}}^{2}-\beta{Z_{in,1}^{i}}^{2} & -2\beta Z_{in,1}^{r}Z_{in,1}^{i} & 0 & J_{+} & \ddots& \ddots& 0 & 0 \\ -2\beta Z_{in,1}^{r}Z_{in,1}^{i} & -3\beta{Z_{in,1}^{i}}^{2}-\beta{Z_{in,1}^{r}}^{2} & -J_{+} & 0 & \ddots& \ddots& 0 & 0 \\ 0 & J_{-} & -3\beta{Z_{in,2}^{r}}^{2}-\beta{Z_{in,2}^{i}}^{2} & -2\beta Z_{in,2}^{r}Z_{in,2}^{i} & \ddots& \ddots& 0 & 0 \\ -J_{-} & 0 & -2\beta Z_{in,2}^{r}Z_{in,2}^{i} & -3\beta{Z_{in,2}^{i}}^{2}-\beta{Z_{in,2}^{r}}^{2} & \ddots& \ddots& 0 & 0 \\ \ddots& \ddots& \ddots& \ddots& \ddots& \ddots& 0 & J_{+} \\ \ddots& \ddots& \ddots& \ddots& \ddots& \ddots& -J_{+} & 0 \\ 0 & 0 & 0 & 0 & 0 & J_{-} & -3\beta{Z_{in,N}^{r}}^{2}-\beta{Z_{in,N}^{i}}^{2} & -2\beta Z_{in,N}^{r}Z_{in,N}^{i} \\ 0 & 0 & 0 & 0 & -J_{-} & 0 & -2\beta Z_{in,N}^{r}Z_{in,N}^{i} & -3\beta{Z_{in,N}^{i}}^{2}-\beta{Z_{in,N}^{r}}^{2} \end{matrix} \right]_{2N\times2N}$

(2)

We find that the real parts of all eigenvalues for Jacobian matrices $\boldsymbol{J}(\boldsymbol{Z}_{\boldsymbol{an}})$ and $\boldsymbol{J}(\boldsymbol{Z}_{\boldsymbol{in}})$ of both anti-phase ($\varepsilon_{an}$) and in-phase ($\varepsilon_{in}$) synchronized states are always negative, as shown in Figures S1c and S1d, indicating that the perturbations around two synchronized states are always attenuated, and both synchronized states are stable.


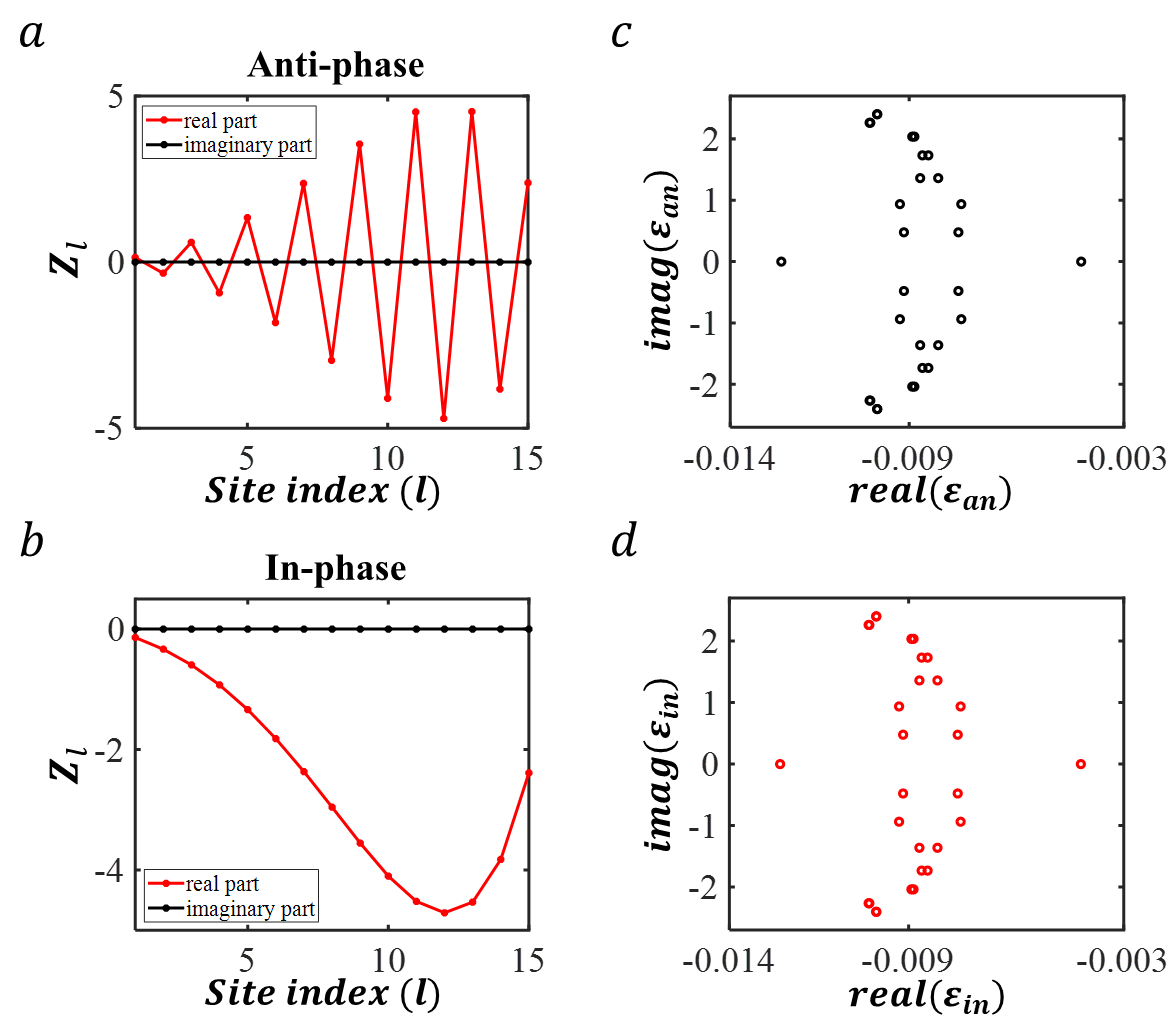


**Figure S1.** (a)-(b). The corresponding spatial distributions of the real and imaginary parts of $\boldsymbol{Z}_{\boldsymbol{an}}$ and $\boldsymbol{Z}_{\boldsymbol{in}}$ (at time marked by dashed lines in Figs. R1a-R1b). (c)-(d). The eigenvalues of the Jacobian matrices $\boldsymbol{J(}\boldsymbol{Z}_{\boldsymbol{an}}\boldsymbol{)}$ and $\boldsymbol{J(}\boldsymbol{Z}_{\boldsymbol{in}}\boldsymbol{)}$ for both anti-phase and in-phase synchronized states.

**Supporting Information 2. Numerical results of the order parameter *R*(*t*) as a function of the lattice length.** It is known that the order parameter $R(t)=\left| \sum_{j=1}^{N} Z_{l}\left( t \right) \right|/N$ can be used to characterize the phase synchronization, being equal to one for phase-locked synchronized states and approaching to zero for non-synchronized states. While, this order parameter is approaching to one for the in-phase non-Hermitian linear synchronized state, but it takes on zero or non-zero value for the anti-phase non-Hermitian linear synchronized state, depending on whether *N* is an even or odd number.

To further illustrate this argument, we present the variation of the order parameter $R(t)$ as a function of the lattice length, as shown in Figure S2a (other parameters are identical to those used in Fig. 1g). Here, twenty random initial states are applied at each lattice length. Figure S2b presents the averaged order parameter $<R\left( t \right)>$ by these random initial states. We can see that Kuramoto order parameter cannot clearly characterize the length region sustaining non-Hermitian skin synchronization. Therefore, we introduce an extended version of the Kuramoto order parameter denoted as $R_{o}=\{max[ R(t)]-min[ R(t)]{\}}_{t>t_{0}}$ ($t_{0}$ being sufficiently large to reach steady states). In this case, we consistently observe that our proposed order parameter approaches to zero for the length region with non-Hermitian global synchronization (both in-phase and anti-phase cases), but exhibits larger values in the non-synchronized region (see Fig. 1g in the main text).


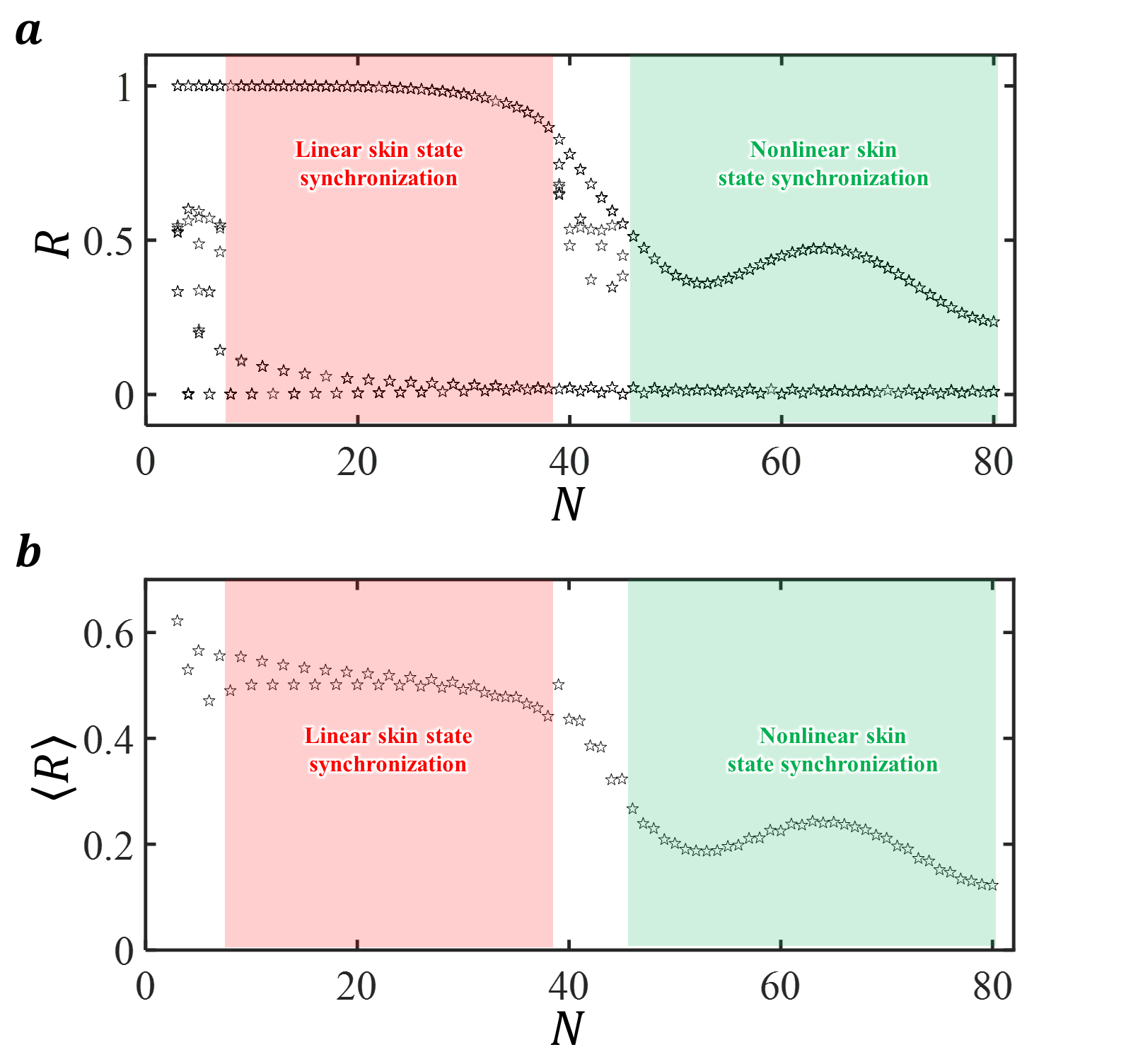


**Figure S2.** (a). Numerical results of Kuramoto order parameter as a function of the lattice length with twenty random initial states. (b). Numerical results of averaged Kuramoto order parameter as a function of the lattice length.

**Supporting Information 3.** **Numerical results of nonlinear dynamics in the system with weak and strong Hatano-Nelson-typed couplings.** In this part, we provide detailed wave dynamics of the model (presented in Fig. 1a in the main text) with different strengths of non-reciprocal couplings. Here, the lattice length is set as *N*=15 and other related parameters are $\omega_{0}=0.1$, $\alpha=5e^{-3},$ and $\beta=5e^{-4}$. Figures S3a and S3b (Figures S3e and S3f) present the wave dynamics and FT frequency spectra of the model with $J_{-}=0.05$ and $J_{+}=1.5$ ($J_{-}=1.4$ and $J_{+}=1.5$), respectively. The corresponding eigenspectra and spatial profiles of eigenmodes for the Hatano-Nelson chain are plotted in Figures S3c and S3d (Figures S3g and S3h). It is clearly shown that the multi-frequency oscillation always appears for both cases with either a weak or a strong non-reciprocity.

If the non-reciprocal strength is too strong ($J_{-}=0.05, J_{+}=1.5)$, all eigenstates are strongly concentrated around the boundary. In this case, the effectiveness on the approximation of linear-eigenstate expansion of nonlinear dynamics is destroyed. This inference can be obtained from the proof by contradiction. If the linear-eigenstate expansion is still hold, a dominated number of nonlinear oscillators located around the opposite side for the skin boundary can exhibit the significant loss effect (with $\alpha\gg\beta{|Z}_{l}|^{2}$). In this case, to maintain the self-sustained oscillation with the balanced loss and gain, the remained oscillator around the skin boundary must possess extremely large amplitudes, which can break the assumption of linear expansion and destroy the non-Hermitian skin synchronization. In this case, the evolution of the system is dominated by the non-linear dynamics, which can be deduced from the corresponding frequency spectra with second- and higher-order frequency components. These higher-order harmonics breaks the non-Hermitian skin synchronization. While, when the non-reciprocal coupling strength is too weak ($J_{-}=1.4, J_{+}=1.5)$, all eigenstates are extended into the whole lattice model and are nearly orthogonal with each other. In this case, the coupling between different eigenstates in the process of nonlinear dynamics is very weak, making the randomly initial state cannot transform into a globally synchronized state.


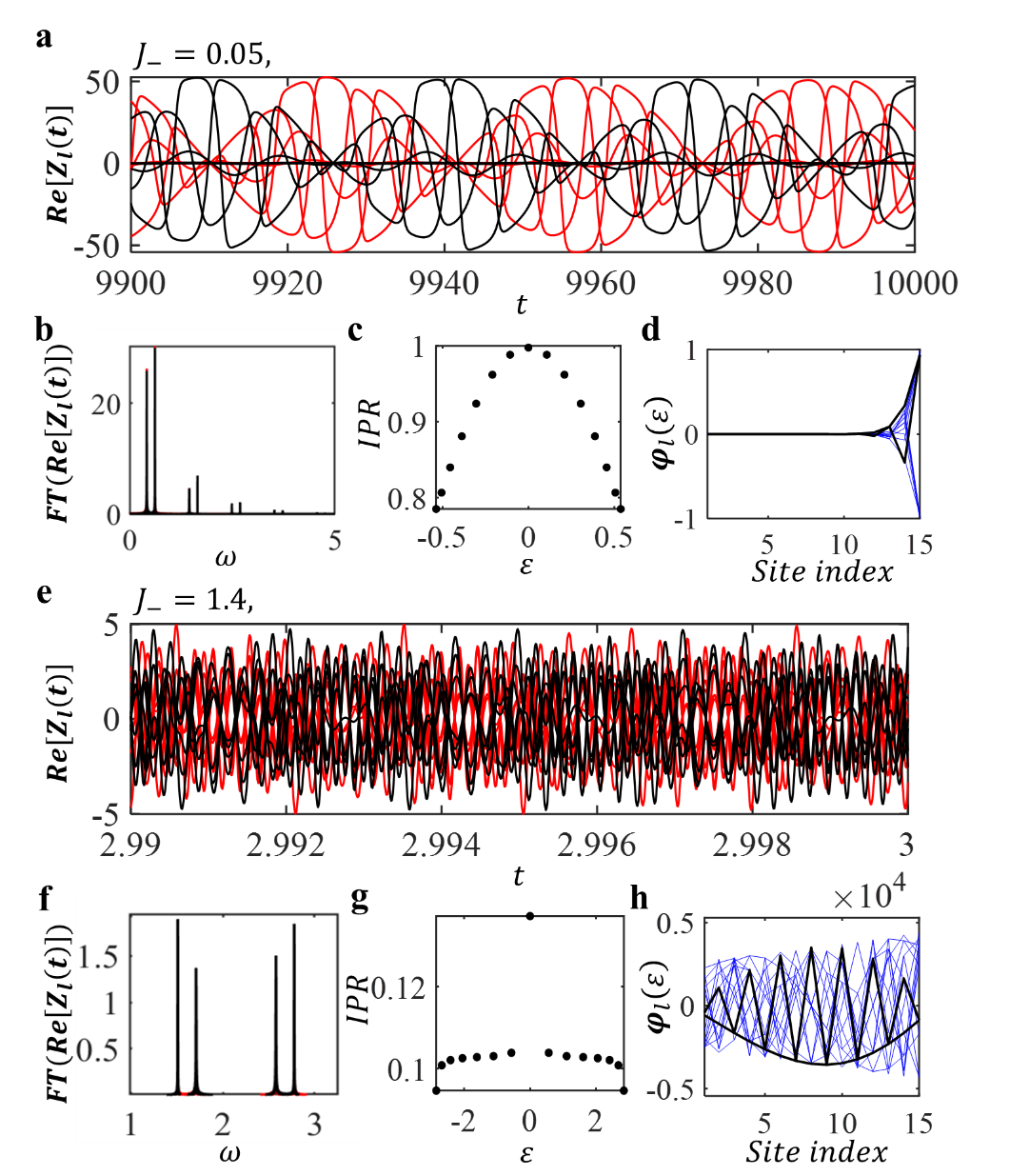


**Figure S3. Numerical results of non-synchronized dynamics with weak and strong non-reciprocal coupling strengths for the nonlinear model with the Hatano-Nelson typed linear coupling.** (a) and (b). Numerical results of the wave dynamics and frequency spectra with $J_{-}=0.05$ and $J_{+}=1.5$. (c) and (d). Eigenspectra and spatial profiles of eigenmodes for the linear part of the model with $J_{-}=0.05$ and $J_{+}=1.5$. (e) and (f). Numerical results of the wave dynamics and frequency spectra with $J_{-}=1.4$ and $J_{+}=1.5$. (c) and (d). Eigenspectra and spatial profiles of eigenmodes for the linear part of the model with $J_{-}=1.4$ and $J_{+}=1.5$. Other related parameters are $\omega_{0}=0.1$, $\alpha=5e^{-3},$ $\beta=5e^{-4}$ and $N=15$.

**Supporting Information 4. Numerical results of nonlinear dynamics with Hatano-Nelson-typed couplings at different lattice lengths.** In this part, we provide numerical results of wave dynamics for the model, whose linear coupling terms correspond to the Hatano-Nelson chain, with different lattice lengths. Other related parameters are set as $\omega_{0}=0.1$, $\alpha=5e^{-3},$ $\beta=5e^{-4}$, $J_{+}=1.5$ and $J_{-}=1.0$.


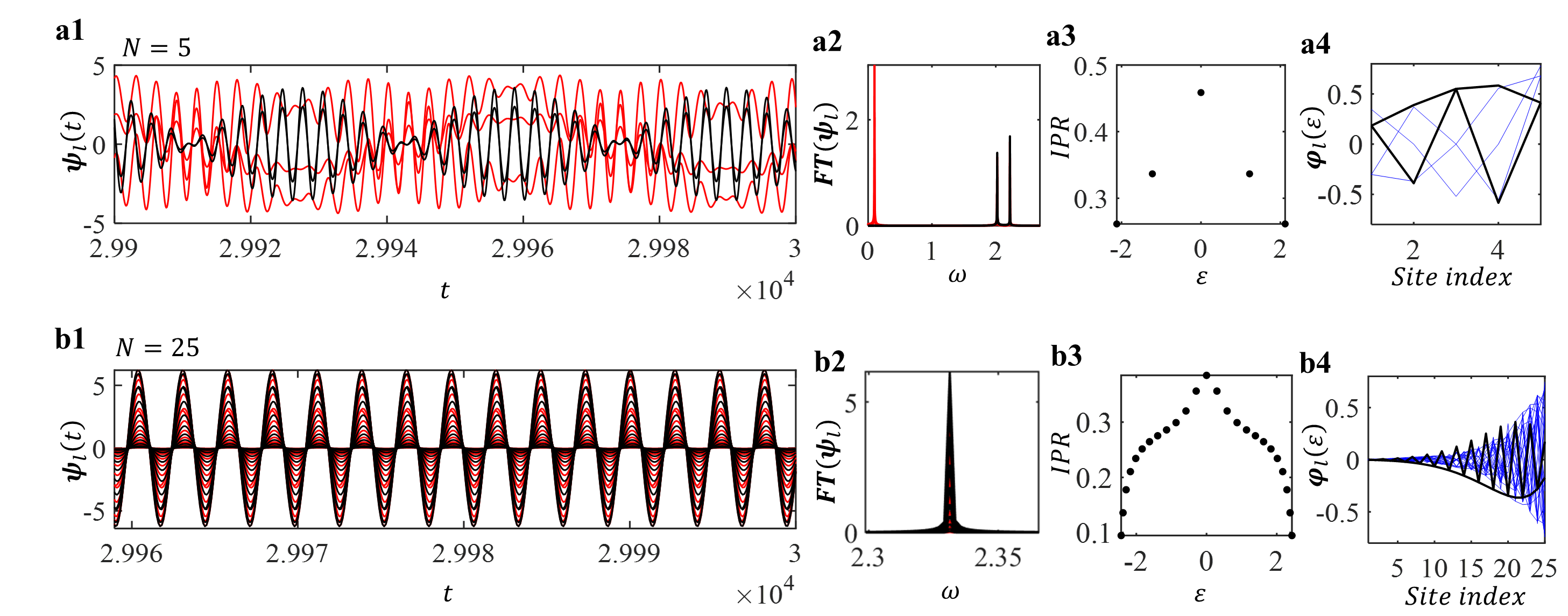


**Figure S4. Numerical results of wave dynamics of the model sustaining non-Hermitian skin synchronization with different lattice lengths.** (a1), (a2) and (b1), (b2). Numerical results of the wave dynamics and FT frequency spectra with $N=5$ and $N=25$. (a3), (a4) and (b3), (b4). Eigenspectra and spatial profiles of linear eigenmodes with $N=5$ and $N=25$.

Figures S4(a1) and S4(a2) (Figures S4(b1) and S4(b2)) present wave dynamics and FT frequency spectra with the lattice length being N=5 (N=25). The corresponding eigenspectra and spatial profiles of linear eigenmodes are plotted in Figures S4(a3) and S4(a4) (Figures S4(b3) and S4(b4)). We can see that all eigenstates of the lattice model with *N*=5 exhibit the extended spatial profiles, and are nearly orthogonal with each other. In this case, the weak coupling between different eigenstates make the system cannot transform into the globally synchronized state in the nonlinear evolution, as shown in Figure S4(a). Differently, in the long-length lattice model with *N*=25, the strong coupling between all initially excited linear eigenmodes can trigger the appearance of non-Hermitian skin ﻿synchronization, as shown in Figure S4(b).

In the transition region (39≤*N*≤45), the order-parameter is significantly increased. To clarify why non-Hermitian skin synchronization cannot be observed within the range of 39≤*N*≤45, we calculate wave dynamics of the lattice model with *N*=41 with random initial states. Figures S5a-S5c present three possible dynamical profiles of $Re[\boldsymbol{Z}_{l}\left( t \right)]$. It is noted that any initial state will transform into one of these three conditions. Figures S5d-S5f and Figures S5g-S5i display the corresponding steady-state profiles (at time marked by dashed lines) and FT frequency spectra. From above numerical results we can see that except for the in-phase and anti-phase non-Hermitian global synchronization (Figures S5a and S5b) with the corresponding steady-state profiles and frequencies being matched to eigen-profiles of minimum-IPR linear skin eigenstates with normalization factors being 35.7, the systematic dynamics with mixed in-phase and anti-phase synchronized states also exists in the intermediate transition region (39≤*N*≤45) (Figure S5c). This is due to the low ratio (~17/41) of lattice sites exhibiting relatively large amplitudes in both in-phase and anti-phase synchronized states (indicated by the red region in Figs. S3d and S3e with $\left| {Re(Z_{l})}/{max[Re\left( Z_{l} \right)]} \right|$>0.05), thereby making the effective coupling between these two synchronized states become relatively weak. Such a weak coupling makes these two synchronized states can coexist in the dynamical evolution of our model.


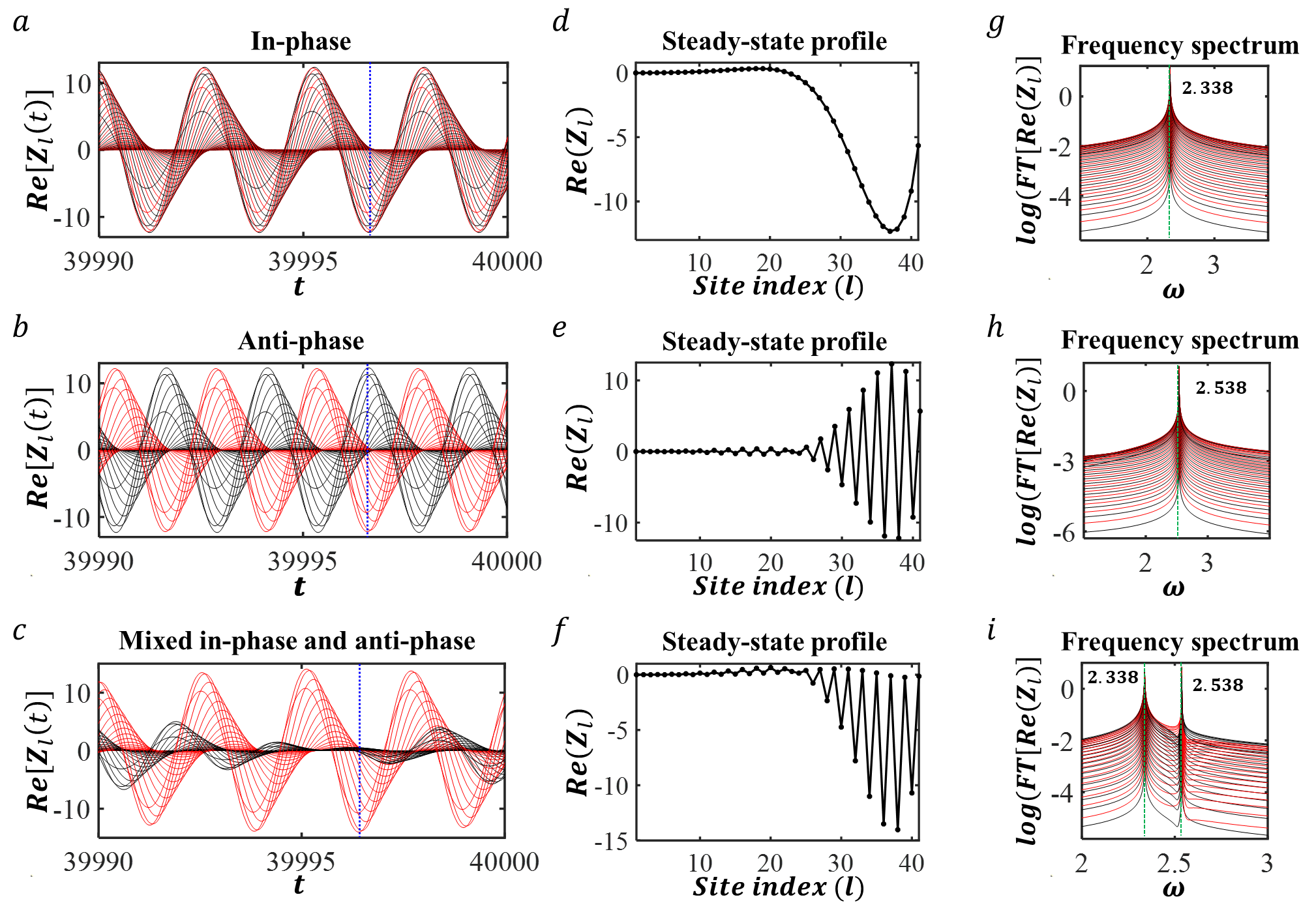


**Figure S5. Numerical results of wave dynamics with N=41.** (a)-(c). Numerical results of three possible wave dynamics with N=41. (d)-(f) and (g)-(i). The corresponding steady-state profiles (at time marked by dashed lines) and FT frequency spectra.

When the lattice length is further increased to *N*>45, the approximation of nonlinear dynamics through linear-eigenstate expansion becomes ineffective, and the evolution is governed by nonlinear eigenstates. Figures S6a and S6b present two possible dynamical profiles $Re[\boldsymbol{Z}_{l}\left( t \right)]$ of synchronized states with *N*=65. Any initial state will transform into one of these two synchronized states. Figures S6c-S6d and Figures S6e-S6f display the corresponding steady-state profiles (at time marked by dashed lines) and FT frequency spectra, being matched to eigen-profiles and eigenenergies of nonlinear eigenstates obtained by Newton-gradient method (See Supporting Information 5). It is noted that, different from the in-phase linear skin synchronized state, the phases of different lattice sites for the nonlinear synchronized state shown in Figures S6a, S6c are not identical. However, for simplicity, we still refer to it as an in-phase non-linear synchronized state. We can see that by further increasing the lattice length, the initial-state-independent non-Hermitian skin synchronization reappears. This arises from the increased ratio (~50/65) of lattice sites exhibiting large wave amplitudes for both in-phase and anti-phase nonlinear skin synchronized states (indicated by red regions in Figs. S7c-d with $\left| {Re(Z_{l})}/{max[Re\left( Z_{l} \right)]} \right|$*>0.05)*, thereby enhancing the effective coupling length between these two synchronized states. The enhanced coupling between these two states ensures the collapse of systematic dynamics into a single synchronized state, which initially dominates the systematic evolution.


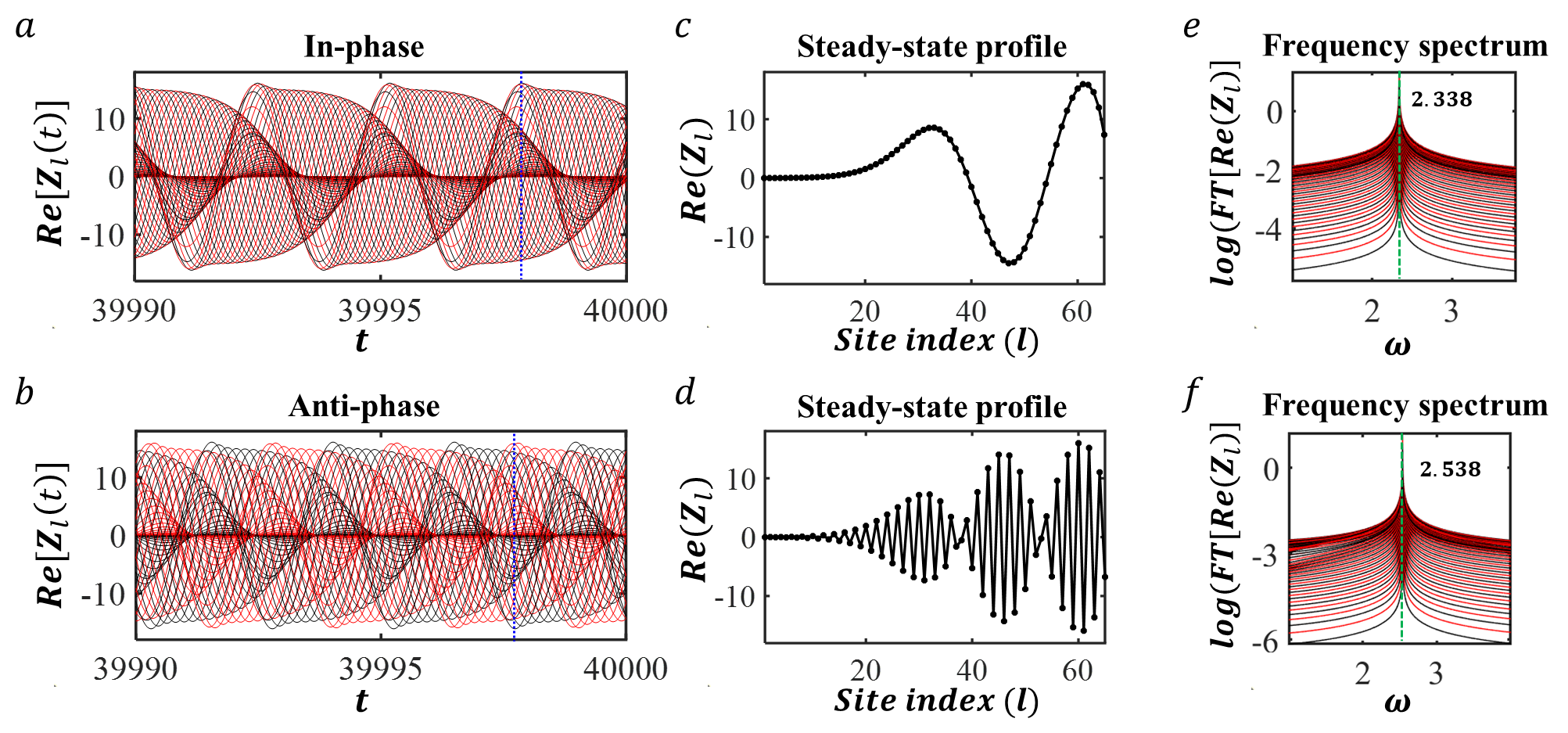


**Figure S6. Numerical results of wave dynamics with N=65.** (a)-(b). Numerical results of two possible wave dynamics with N=65. (c)-(d) and (e)-(f). The corresponding steady-state profiles (at time marked by dashed lines) and FT frequency spectra.

To further compare the effective coupling between in-phase and anti-phase synchronized states in different length regions, we present their spatial profiles with N=15, 25, 38 (linear skin synchronization), N=41 (the transition region) and N=48, 65 (nonlinear skin synchronization), as shown in Figures S7a-S7f, respectively. Red blocks denote lattice sites possessing large amplitudes of both in-phase and anti-phase synchronized states with $\left| {Re(Z_{l})}/{max[Re\left( Z_{l} \right)]} \right|$*>*$Z_{t}$*,* that can contribute to the effective coupling between two synchronized states during the self-excited oscillation. Here, we set $Z_{t}=0.05$. It is shown that both linear (7<*N*≤38) and nonlinear (*N*>45) skin synchronized states possess the larger ratio of lattice sites satisfying $\left| {Re(Z_{l})}/{max[Re\left( Z_{l} \right)]} \right|$*>*$Z_{t}$ (corresponding to large amplitudes of in-phase and anti-phase synchronized states) than that of the transition region (39≤*N*≤45). Thus, due to the relatively weak coupling between two synchronized states in the intermediate transition region of *N*=41, the in-phase and anti-phase synchronized states can coexist in the dynamical evolution. It is noted that our conclusion regarding the requirement of a longer coupling length between anti-phase and in-phase synchronized states for non-Hermitian skin ﻿synchronization remains unaffected by the chosen of the threshold $Z_{t}$.


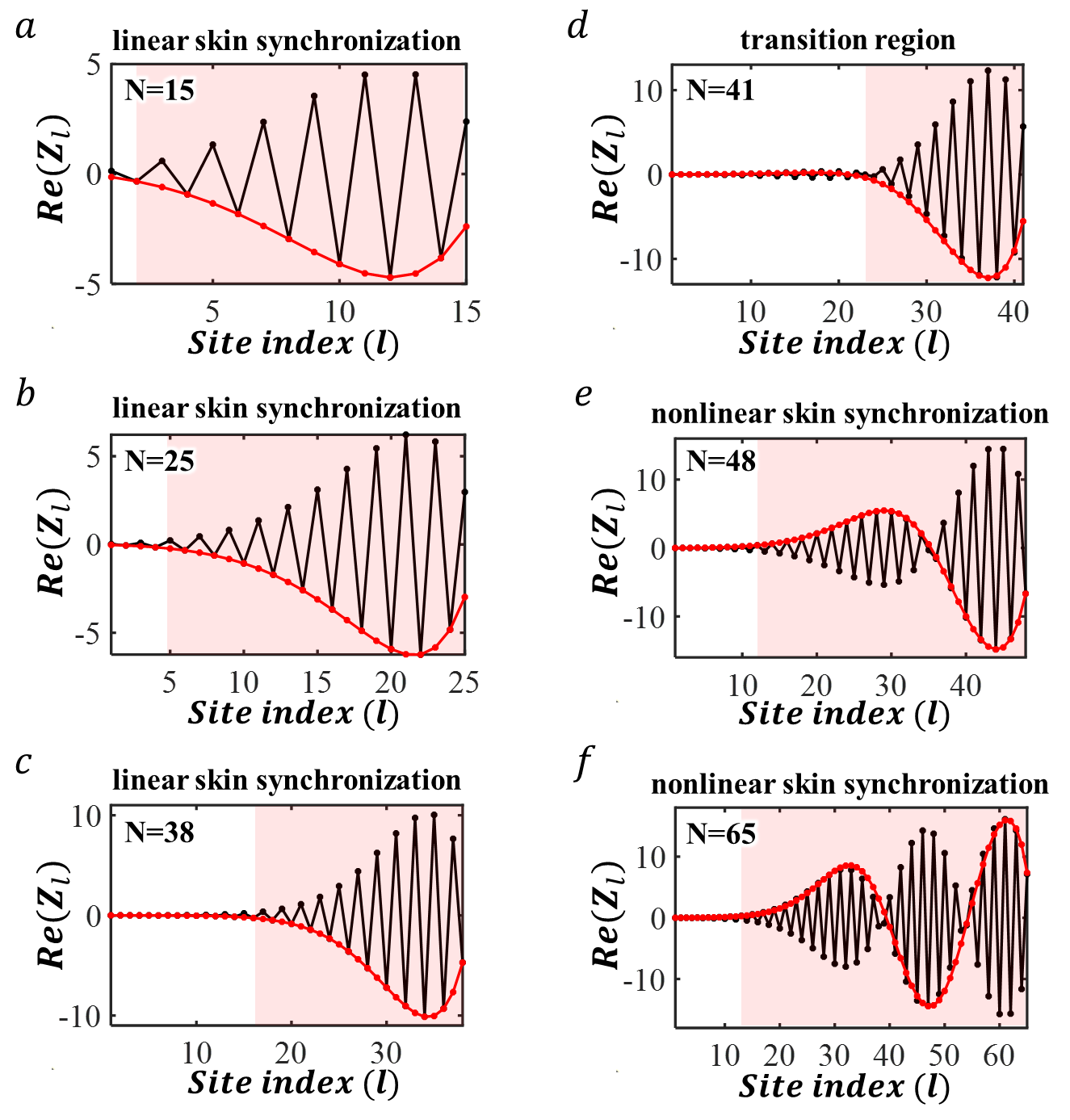


**Figure S7. Numerical results of steady-state spatial profiles with different lattice lengths.**  (a)-(f). Spatial profiles of in-phase and anti-phase synchronized states with N=15, 25, 38 (linear skin synchronization), N=41 (transition region), and N=48, 65 (nonlinear skin synchronization), respectively.

**Supporting Information 5.** **Nonlinear eigenstates of the model with Hatano-Nelson-typed couplings based on Newton-gradient method.** In this part, we provide numerical results on nonlinear eigenstates of the lattice model, which sustains non-Hermitian skin synchronization, based on Newton-gradient method. At first, we give details on the Newton-gradient method. By substituting the harmonic wave solution $\boldsymbol{Z(t)=Z}\boldsymbol{e}^{\boldsymbol{-i\varepsilon t}}$ into the time-dependent nonlinear Schrodinger’s equation (Eq. (1) in the main text), we obtain the steady-state nonlinear eigenequation as

$-i\varepsilon Z_{l}=\left( i\omega_{0}+\alpha-\beta\left| Z_{l} \right|^{2} \right)Z_{l}-i{(J}_{+}Z_{l+1}+J_{-}Z_{l-1})$. (3)

We express Eq. (3) in the form of

$f_{l}(\boldsymbol{Z})=-(\varepsilon+\omega_{0})Z_{l}+i\alpha Z_{l}-i\beta\left| Z_{l} \right|^{2}Z_{l}+{(J}_{+}Z_{l+1}+J_{-}Z_{l-1})$, (4)

where $\boldsymbol{f}=[f_{1},\ldots,f_{N}]$ becomes a null vector with $\boldsymbol{\varepsilon}$ and ${\boldsymbol{Z}=[Z}_{1},\ldots,Z_{N}]$ being the eigenenergy and the associated eigenstate of the nonlinear eigenequation. We separately express the real and imaginary parts of $f_{l}(\boldsymbol{Z})$ as

$Re(f_{l}(\boldsymbol{Z}))=-\varepsilon^{r}Z_{l}^{r}-i\varepsilon^{i}*iZ_{l}^{i}-\omega_{0}^{r}Z_{l}^{r}-i\omega_{0}^{i}*iZ_{l}^{i}+i\alpha*iZ_{l}^{i}-i\beta*i{Z_{l}^{r}}^{2}Z_{l}^{i}$

$-i\beta*i{Z_{l}^{i}}^{3}+J_{+}^{r}*Z_{l}^{r}+iJ_{+}^{i}*iZ_{l}^{i}+J_{-}^{r}*Z_{l}^{r}+iJ_{-}^{i}*iZ_{l}^{i}$ (5)

$Im\left( f_{l}\left( \boldsymbol{Z} \right) \right)=-\varepsilon^{r}*iZ_{l}^{i}-i\varepsilon^{i}Z_{l}^{r}-\omega_{0}^{r}*iZ_{l}^{i}-i\omega_{0}^{i}Z_{l}^{r}+i\alpha*Z_{l}^{r}-i\beta*{Z_{l}^{r}}^{3}$

$-i\beta*{Z_{l}^{i}}^{2}Z_{l}^{r}+J_{+}^{r}*iZ_{l}^{i}+iJ_{+}^{i}*Z_{l}^{r}+J_{-}^{r}*iZ_{l}^{i}+iJ_{-}^{i}*Z_{l}^{r}$ (6)

where $Re(f_{l}(\boldsymbol{Z}))$ and $Im(f_{l}(\boldsymbol{Z}))$ are real and imaginary parts of $f_{l}(\boldsymbol{Z})$. $Z_{l}^{r}$ and $Z_{l}^{i}$ correspond to the real and imaginary parts of the complex amplitude $Z_{l}$. $\varepsilon^{r}$ and $\varepsilon^{i}$ are the real and imaginary parts of the eigenenergy $\boldsymbol{\varepsilon}$. Eqs. (5) and (6) can be combined in the matrix form of $\boldsymbol{v}=\boldsymbol{Fu}$ with ${v=[Re\left( f_{1}\left( \boldsymbol{Z} \right) \right),Im\left( f_{1}\left( \boldsymbol{Z} \right) \right),\ldots,Re\left( f_{N}\left( \boldsymbol{Z} \right) \right),Im\left( f_{N}\left( \boldsymbol{Z} \right) \right)]}^{T}$ and ${u=[Z_{1}^{r},Z_{1}^{i},\ldots Z_{N}^{r},Z_{N}^{i},\varepsilon^{r},\varepsilon^{i}]}^{T}$ being the $2N$ by 1 and $2N+2$ by 1 matrixes, respectively. $\boldsymbol{F}$ is the $2N$ by $2N+2$ Jacobian matrix used in our Newton-gradient method, and the matrix element $F_{(m,p)}$ is expressed as

$F_{(m,p)}=\frac{\partial v_{m}(Z)}{\partial u_{p}}$ (7)

with $m\propto[1, \ldots,2N]$ and $p\propto[1, \ldots,2N+2]$. Based on the Jacobian matrix, we can iteratively update the nonlinear solution as

$u_{new}=u_{old}-F^{-1}v$ (8)

where $u_{old}$ and $u_{new}$ represent the input and updated solutions, containing both the eigenenergy and eigenstate. By literately implement Eq. (8), we can obtain the final nonlinear eigenstate and eigenenergy, which satisfy the nonlinear Schrodinger’s equation. It is important to note that the suitable initial state used in Newton-gradient method can play a key role in solving the nonlinear eigenequation.


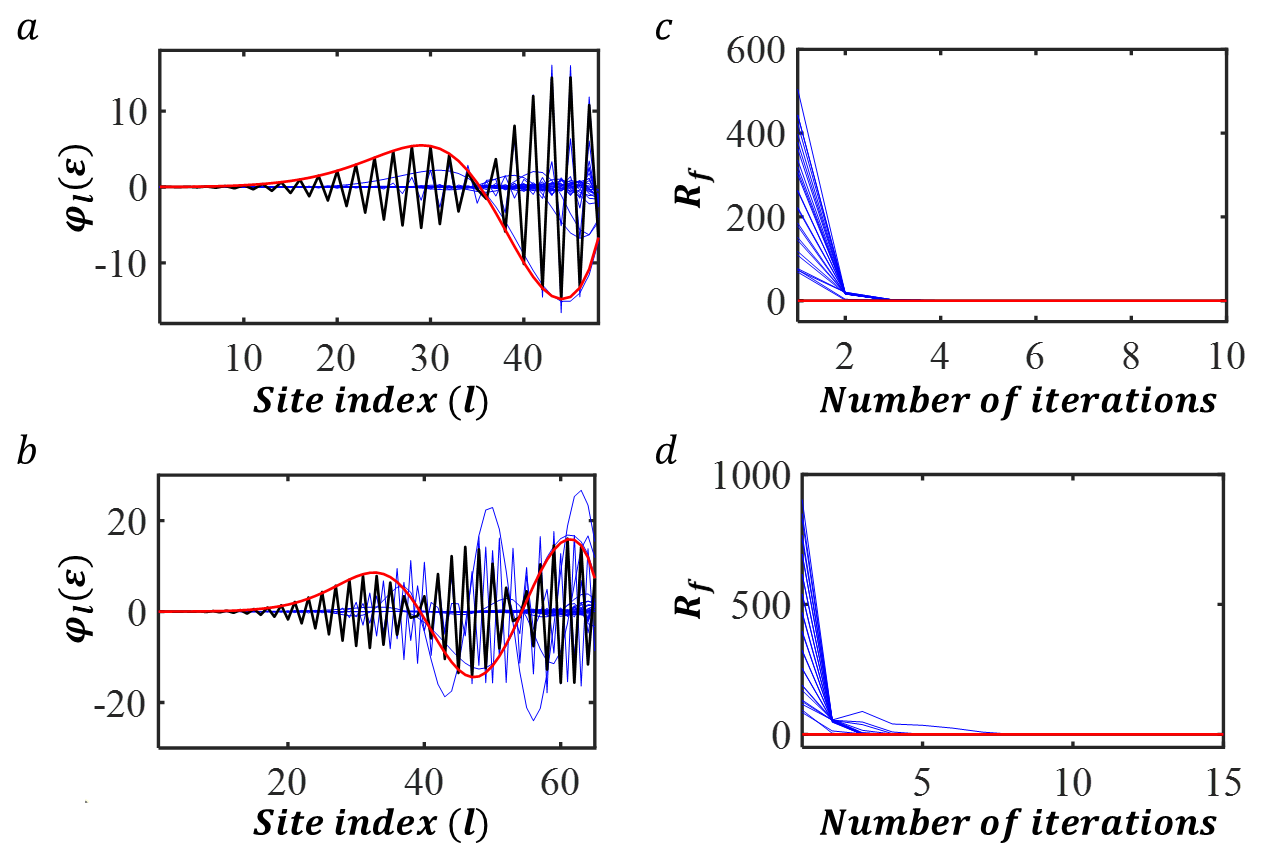


**Figure S8. Numerical results on nonlinear eigenstates of the model with non-Hermitian skin synchronization.** (a) and (b) The calculated nonlinear eigenstates for the lattice model with N=48 and 65. (c) and (d). The convergence graphs for different nonlinear states with N=48 and 65.

Figures S8a and S8b present the calculated nonlinear eigenstates with N=48 and 65, respectively. Here, we show several reprehensive nonlinear eigenstates, where the red (black) lines present two eigenstates with the minimum localization strength, being matched to anti-phase and in-phase synchronized states (see Figure S6). Other parameters are identical to that used in Fig. 1g of the main text. The corresponding convergence graphs, defining as $R_{f}=\sum_{l=1}^{N} \left| Re\left( f_{l}\left( \boldsymbol{Z} \right) \right) \right|$, are plotted in Figures S8c and S8d.

**Supporting Information 6.** **The relationship between IPRs of non-Hermitian SSH model and order-parameters.** In this part, we discuss the relationship between IPRs of linear eigenstates of non-Hermitian SSH model and the order-parameter of nonlinear dynamics. As shown in Figures S9a-S9d, we calculate eigenspectra accomplished with *IPRs* of all eigenstates for the non-Hermitian SSH model (Fig. 3a in the main text) with the reciprocal intercell coupling being $J=0.2$, $0.35,$ $0.5$ and $1.0$, respectively. Other system parameters are set as $N=25$, $J_{+}=0.56$ and $J_{-}=0.1$. Additionally, the corresponding spatial profiles of all eigenstates are presented in right charts, where two non-Hermitian skin states with the smallest *IPR* and a midgap topological state are highlighted by black/red and green lines. It is clearly shown that the spatial profile of mid-gap topological state can be significantly influenced by the intercell coupling. In particular, it is localized on the same boundary with other skin states with the relatively small values of $J=0.2$ and $0.35$. Moreover, such a midgap topological state can also localize on the opposite boundary with respect to the non-Hermitian skin states with $J=1.0$. And, it can also completely extend into the entire bulk region with $J=0.5$.


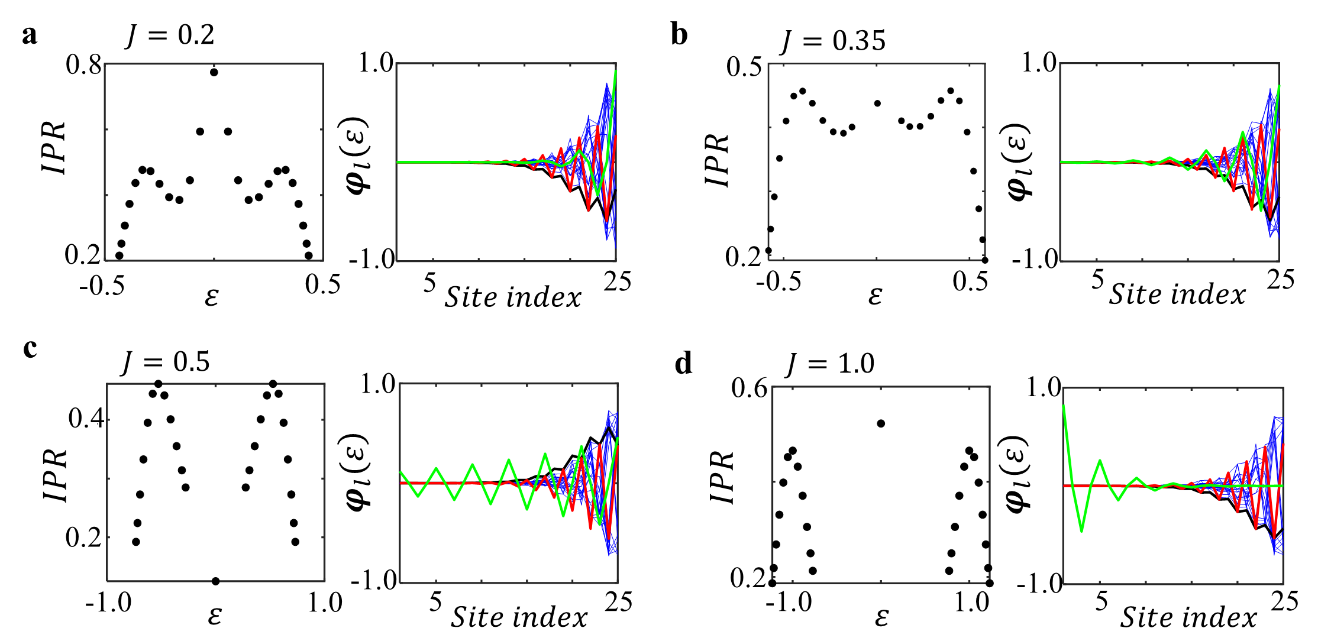


**Figure S9. The influence of the reciprocal inter-cell coupling on the properties of midgap topological states.** (a)-(d). Numerical results of eigenspectra, the spatial profile and *IPR* of each eigenstate of four linear Hamiltonians with different intercell coupling strengths, which equal to $J=0.2$, $J=0.35$, $J=0.5$ and $J=1.0$, respectively.

Then, we clarify the relationship between the phase transition of nonlinear dynamics and *IPR*s of all linear eigenstates. The top (bottom) chart in Figure S10 presents the variation of order-parameter (IPRs of all eigenmodes) as a function of the reciprocal coupling strength $J$. Other system parameters are set as $\omega_{0}=0.1$, $\alpha=5e^{-4},$ $\beta=5e^{-5}$ and $J_{+}=0.56$, $J_{-}=0.1$ and $N=25$. The red dash line marks the critical point for the IPR of midgap topological state becoming the minimum value of all eigenstates. It is clearly shown that the system can evolve into the topological global synchronization with the value of $J$ exceeding such a critical point. While, it is worth noting that the significantly large coupling strength between the midgap topological state and other skin states is a prerequisite for the appearance of topological global synchronization except for the minimum value of IPR of the midgap topological state. Both of these two requirements determine the range (the red region) of intercell coupling strength $J$ in the model sustaining the topological global synchronization. The green dash line marks the critical point for the IPR of the midgap topological state becoming the maximum value. The strong couplings between the midgap topological state and non-Hermitian skin states can appear in the process of nonlinear dynamics when the IPR of the midgap topological state is merged into the IPR continuum of all skin states (with the intercell coupling in the range between red and green dash lines). In this case, the wave amplitudes of all site oscillators are significantly enlarged, making the effectiveness on the approximation of linear-eigenstate expansion of nonlinear dynamics be destroyed. We find that the nonlinear-eigenstate dominated non-Hermitian synchronization can also appear under a suitable value of the intercell coupling strength $J$ (the green region). It is worth noting that although the synchronized oscillation frequency still equals to $\omega=0.1$, it does not correspond to the midgap linear topological state, but a nonlinear eigenvalue of the associated nonlinear eigenmode. The purple dash line marks the critical point for the appearance of non-Hermitian skin synchronization. Across such a critical point, the IPR of a skin state is significantly increased towards to that of the midgap topological state. In this case, the enhanced coupling between the midgap topological state and non-Hermitian skin states can be induced, ensuring that the midgap topological state can evolve into the non-Hermitian skin state with a minimum IPR.


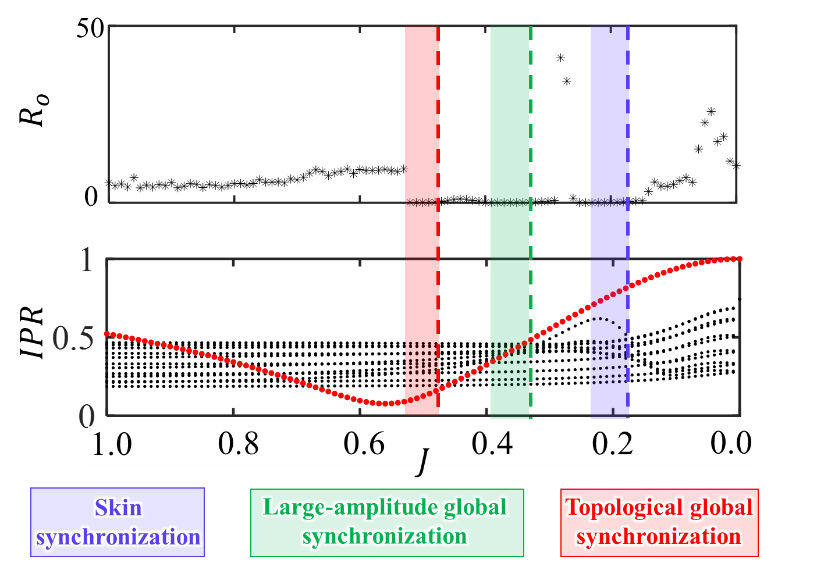


**Figure S10. Numerical results of order-parameter and IPRs of the system as a function of the reciprocal intercell coupling strength.** The top and bottom charts present the variations of the order-parameter and IPRs of all linear eigenstates as a function of the reciprocal coupling strength $J$. Other parameters are set as $\omega_{0}=0.1$, $\alpha=5e^{-4},$ $\beta=5e^{-5}$, $J_{+}=0.56$, $J_{-}=0.1$ and $N=25$.

**Supporting Information 7.** **Nonlinear eigenstates of the model with non-Hermitian SSH-typed couplings based on Newton-gradient method.** In this part, we provide numerical results on nonlinear eigenstates of the model, which sustains non-Hermitian topological synchronization, based on Newton-gradient method. Figure S11a present the calculated nonlinear eigenstates with J=0.35. Other parameters are identical to that used in Fig. 3j of the main text. The red lines present results with the initial state used in Newton-gradient method being the steady state presented in Fig. 3(g3) in the main text. The corresponding convergence graph is plotted in Figure S11b.


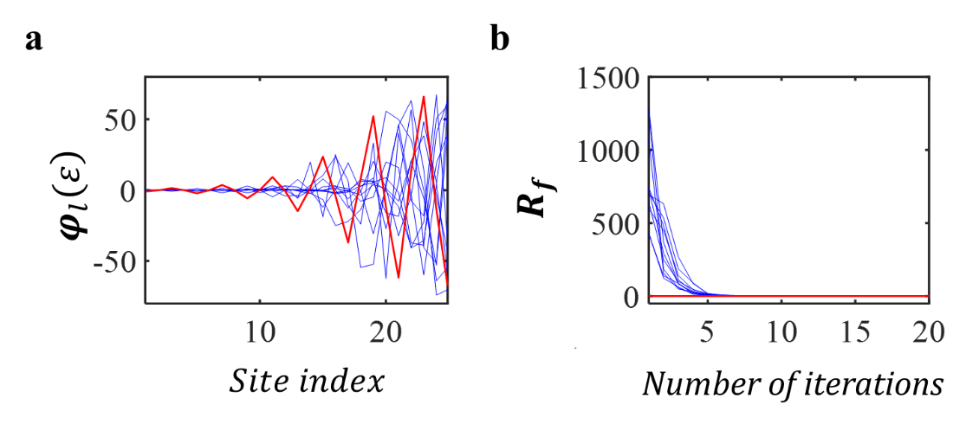


**Figure S11. Numerical results on nonlinear eigenstates of the model with non-Hermitian topological global synchronization.** (a) The calculated nonlinear eigenstates for the lattice model with J=0.35. (b) The results of the convergence graph for different nonlinear states with J=0.35.

**Supporting Information 8. The robustness of non-Hermitian topological global ﻿synchronization.** To clarify the robustness of non-Hermitian topological global synchronization, we introduce random disorder to the self-oscillated frequency of each oscillator with $\omega=\omega_{0}+[-W,W]$, where $W$ quantifies the strength of disorder. Other parameters are identical with those used in Fig. 3h in the main text, where the topological global synchronization exists in the clean limit. Figures S12(a1) and S12(b1) depict the wave dynamics with disorder strengths being *W*=0.1 and 0.5, respectively. The corresponding FT frequency spectra and the steady-state spatial profiles are shown in Figures S12(a2) and S12(b2) for *W*=0.1 and Figures S12(a3) and S12(b3) for *W*=0.5, respectively. It is shown that non-Hermitian topological global synchronization persists when the disorder strength is relatively weak (*W*=0.1). In this case, the oscillation frequency and steady-state profile still match to the eigenenergy and eigenstate of the midgap topological state with disorder. It is worth noting that there are non-zero amplitudes at *B*-type sublattices in the steady-state spatial profile, being consistent with the linear topological state with disorder. Under a much stronger disorder strength (*W*=0.5), wave amplitudes of nonlinear oscillators become significantly enlarged. In this case, the assumption of linear-eigenstate expansion of nonlinear dynamics becomes ineffective, thereby destroying the non-Hermitian topological global synchronization.


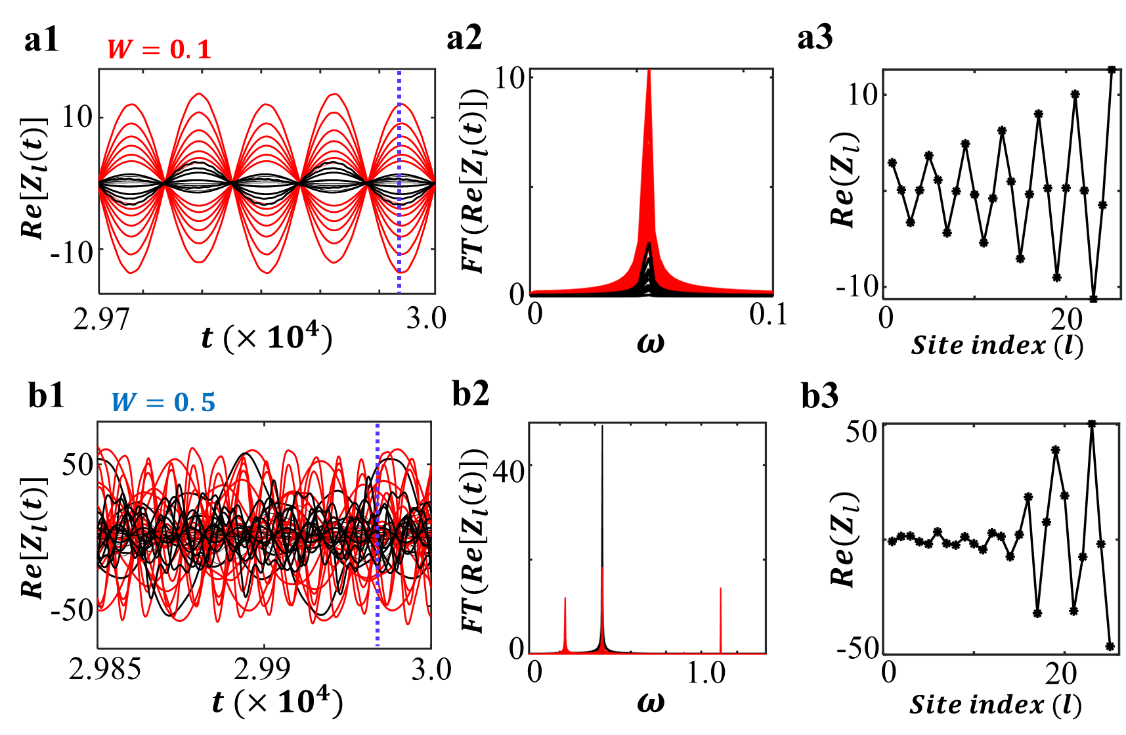


**Figure S12. The robustness of non-Hermitian topological global ﻿synchronization.** (a1), (b1) and (a2), (b2). The wave dynamics and FT frequency spectra of all oscillators in the system with disorder strengths being W=0.1 and 0.5, where the system exhibit the extended topological global synchronization in the clean limit. The corresponding spatial profiles of steady states are plotted in (a3) and (b3). Here, the systematic parameters are set as N=15, $J=0.5$, $J_{+}=0.56$ and $J_{-}=0.1$.

**Supporting Information 9. Numerical results of nonlinear dynamics with non-Hermitian SSH-typed couplings at different lattice lengths.** In this part, we provide numerical results of wave dynamics for the system with the linear coupling being the non-Hermitian SSH model. Here, the related parameters are set identical to that used in Fig. 3k in the main text. Figures S13(a1) and S13(b1) present wave dynamics with the lattice length being N=11 and 31. Figures S13(a2) and S13(b2) and Figures S13(a3) and S13(b3) display the corresponding FT frequency spectra and steady-state profiles with *N*=11 and 31, respectively.

In the short lattice-length region with *N*=11, the system dynamics exhibit the small-valued wave amplitudes, and are dominated by the linear eigenstates of the non-Hermitian SSH model. In this case, the system can exhibit the non-Hermitian topological global synchronization, being consistent to the near zero-valued order-parameter in the red region of Fig. 3k in the main text. While, in the long lattice-length region with N=31, the system dynamics possess large wave amplitudes, where the approximation of linear-eigenstate expansion on the system dynamics is disrupted and the nonlinear eigenstates govern the system dynamics. It is shown that non-Hermitian global synchronization with large amplitudes appears with N=31, being consistent to the near zero-valued order-parameter in the green region of Fig. 3k in the main text.


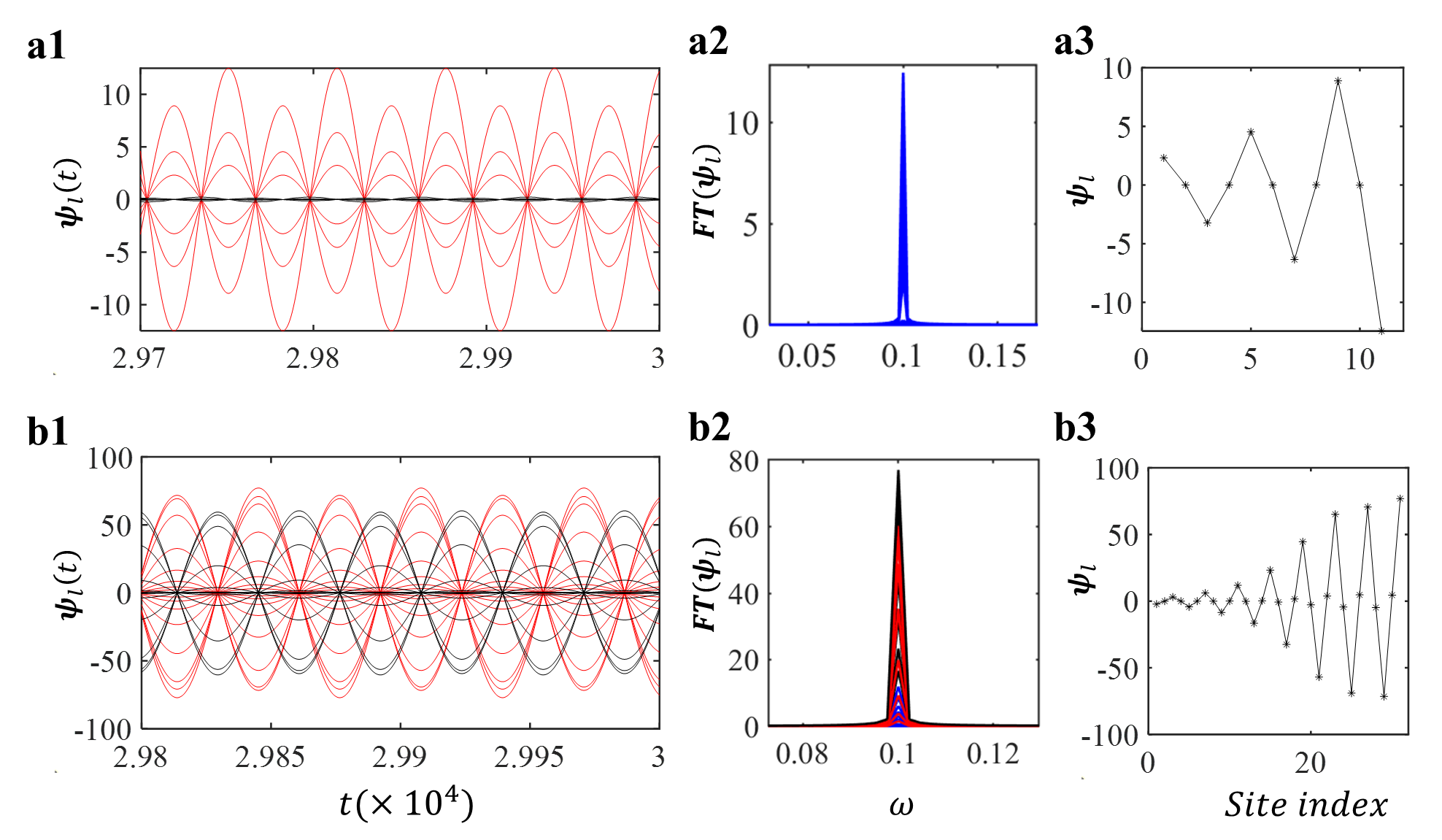


**Figure S13. Numerical results of wave dynamics in the non-Hermitian SSH model with different lattice lengths.** (a1) and (b1). Numerical results of the wave dynamics with N=11 and 31. (a2)-(a3) and (b2)-(b3). Numerical results of FT frequency spectra and steady-state profile with N=11 and 31. Other related parameters are $\omega_{0}=0.1$, $\alpha=5e^{-4},$ $\beta=5e^{-5}$, $J=0.4$, $J_{+}=0.56$ and $J_{-}=0.1$.

**Supporting Information 10. The correspondence between Chua diode and Stuart-Landau oscillator.** In this part, we provide details on the correspondence between Chua diode and Stuart-Landau oscillator. Figure S14 presents the schematic diagram of Chua diode, which is composed by two multipliers and an INIC. Based on the relationship between the input and output voltages of the first and second multipliers, we can obtain two relationships of $V_{2}=\frac{{V_{i,1}}^{2}}{10}$ and $V_{3}=\frac{V_{i,1}*V_{2}}{10}\frac{{R_{4}+R}_{5}}{R_{4}}$. Combing these two terms, we have $V_{3}=\frac{R_{4}+R_{5}}{100R_{4}}{V_{i,1}}^{3}$. By applying Kirchhoff’s law on the circuit node of $V_{i,1}$, we obtain the following equation as

$\frac{dV_{i,1}}{dt}=\frac{1}{{CR}_{3}}V_{i,1}-\frac{R_{4}+R_{5}}{100CR_{3}R_{4}}{V_{i,1}}^{3}$. (9)

We can see that Supplementary Eq. (3) possesses the same form to the dynamical equation of Stuart-Landau oscillator with effective parameters being $\alpha=\frac{1}{{CR}_{3}}$ and $\beta=\frac{R_{4}+R_{5}}{100CR_{3}R_{4}}$. It is noted that the oscillation frequency $\omega_{0}$ is realized by the non-reciprocal resistance $\pm R_{w}$ as deviated below.


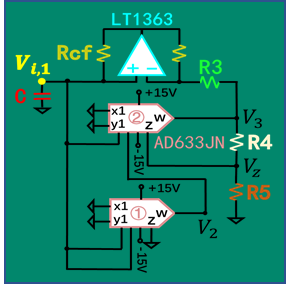


**Figure S14. The schematic diagram of the Chua diode, which is composed of two multipliers and a non-reciprocal resistance.**

**Supporting Information 11. The derivation of eigenequation for the non-Hermitian ﻿**﻿**synchronized circuit.** In this part, we give details for the derivation of eigenequation for non-Hermitian ﻿**﻿**﻿synchronized electric circuits. Firstly, we consider the electric circuit sustaining non-Hermitian skin ﻿﻿synchronization**.** Here, each lattice site possesses four circuit nodes, and voltages at these four circuit nodes in the *i*th unit should be written as $V_{i}={[V}_{i,1},V_{i,2},V_{i,3}{,V_{i,4}]}^{T}$. Carrying out ﻿Kirchhoff’s law on four circuit nodes (there is no external source), we obtain the following equations as:

$-C\frac{d}{dt}\left[ \begin{matrix} \begin{matrix} V_{i,1} \\ V_{i,2} \end{matrix} \\ V_{i,3} \\ V_{i,4} \end{matrix} \right]{=({-R}_{1}}^{-1}+{R_{2}}^{-1})\left[ \begin{matrix} V_{i,1}-V_{i+1,2} \\ V_{i,2}-V_{i+1,3} \\ \begin{matrix} V_{i,3}-V_{i+1,4} \\ V_{i,4}-V_{i+1,1} \end{matrix} \end{matrix} \right]+({R_{1}}^{-1}+{R_{2}}^{-1})\left[ \begin{matrix} V_{i,1}-V_{i-1,4} \\ V_{i,2}-V_{i-1,1} \\ \begin{matrix} V_{i,3}-V_{i-1,2} \\ V_{i,4}-V_{i-1,3} \end{matrix} \end{matrix} \right]+(-R_{a})^{-1}\boldsymbol{I}_{4\times4}\left[ \begin{matrix} V_{i,1} \\ V_{i,2} \\ \begin{matrix} V_{i,3} \\ V_{i,4} \end{matrix} \end{matrix} \right]$

$+\left[ \begin{matrix} \begin{matrix} 0 & -(R_{w})^{-1} \\ (R_{w})^{-1} & 0 \end{matrix} & \begin{matrix} 0 & (R_{w})^{-1} \\ -(R_{w})^{-1} & 0 \end{matrix} \\ \begin{matrix} 0 & (R_{w})^{-1} \\ -(R_{w})^{-1} & 0 \end{matrix} & \begin{matrix} 0 & -(R_{w})^{-1} \\ (R_{w})^{-1} & 0 \end{matrix} \end{matrix} \right]\left[ \begin{matrix} V_{i,1} \\ V_{i,2} \\ \begin{matrix} V_{i,3} \\ V_{i,4} \end{matrix} \end{matrix} \right]+(-\frac{1}{R_{3}}+\frac{R_{4}+R_{5}}{100R_{3}R_{4}}[\left| V_{i,1} \right|^{2},\left| V_{i,2} \right|^{2},\left| V_{i,3} \right|^{2},\left| V_{i,4} \right|^{2}])\left[ \begin{matrix} V_{i,1} \\ V_{i,2} \\ \begin{matrix} V_{i,3} \\ V_{i,4} \end{matrix} \end{matrix} \right]$ (10)

Performing the diagonalization of Supplementary Eq. (10) with a unitary transformation

$F=\frac{1}{\sqrt{4}}\left[ \begin{matrix} \begin{matrix} 1 & 1 \\ 1 & e^{i2\pi/4} \end{matrix} & \begin{matrix} 1 & 1 \\ e^{i4\pi/4} & e^{i6\pi/4} \end{matrix} \\ \begin{matrix} 1 & e^{i4\pi/4} \\ 1 & e^{i6\pi/4} \end{matrix} & \begin{matrix} e^{i8\pi/4} & e^{i12\pi/4} \\ e^{i12\pi/4} & e^{i18\pi/4} \end{matrix} \end{matrix} \right]$, (11)

it becomes

$$-C\frac{d}{dt}\left[ \begin{matrix} \begin{matrix} V_{i,\to} \\ V_{i,\uparrow} \end{matrix} \\ V_{i,\leftarrow} \\ V_{i,\downarrow} \end{matrix} \right]={(R_{2}}^{-1}-{R_{1}}^{-1}+{R_{1}}^{-1}+{R_{2}}^{-1}-{R_{a}}^{-1})\left[ \begin{matrix} V_{i,\to} \\ V_{i,\uparrow} \\ \begin{matrix} V_{i,\leftarrow} \\ V_{i,\downarrow} \end{matrix} \end{matrix} \right]$$

$$+(-\frac{1}{R_{3}}+\frac{R_{4}+R_{5}}{100R_{3}R_{4}}[\left| V_{i,\to} \right|^{2},\left| V_{i,\uparrow} \right|^{2},\left| V_{i,\leftarrow} \right|^{2},\left| V_{i,\downarrow} \right|^{2}])\left[ \begin{matrix} V_{i,\to} \\ V_{i,\uparrow} \\ \begin{matrix} V_{i,\leftarrow} \\ V_{i,\downarrow} \end{matrix} \end{matrix} \right]-{({-R}_{1}}^{-1}+{R_{2}}^{-1})\left[ \begin{matrix} \begin{matrix} 1 & 0 \\ 0 & e^{\frac{i\pi}{2}} \end{matrix} & \begin{matrix} 0 & 0 \\ 0 & 0 \end{matrix} \\ \begin{matrix} 0 & 0 \\ 0 & 0 \end{matrix} & \begin{matrix} e^{i\pi} & 0 \\ 0 & e^{-\frac{i\pi}{2}} \end{matrix} \end{matrix} \right]\left[ \begin{matrix} V_{i+1,\to} \\ V_{i+1,\uparrow} \\ \begin{matrix} V_{i+1,\leftarrow} \\ V_{i+1,\downarrow} \end{matrix} \end{matrix} \right]$$

$-({R_{1}}^{-1}+{R_{2}}^{-1})\left[ \begin{matrix} \begin{matrix} 1 & 0 \\ 0 & e^{-i\pi/2} \end{matrix} & \begin{matrix} 0 & 0 \\ 0 & 0 \end{matrix} \\ \begin{matrix} 0 & 0 \\ 0 & 0 \end{matrix} & \begin{matrix} e^{i\pi} & 0 \\ 0 & e^{i\pi/2} \end{matrix} \end{matrix} \right]\left[ \begin{matrix} V_{i-1,\to} \\ V_{i-1,\uparrow} \\ \begin{matrix} V_{i-1,\leftarrow} \\ V_{i-1,\downarrow} \end{matrix} \end{matrix} \right]-j\left[ \begin{matrix} \begin{matrix} 0 & 0 \\ 0 & 2(R_{w})^{-1} \end{matrix} & \begin{matrix} 0 & 0 \\ 0 & 0 \end{matrix} \\ \begin{matrix} 0 & 0 \\ 0 & 0 \end{matrix} & \begin{matrix} 0 & 0 \\ 0 & -2(R_{w})^{-1} \end{matrix} \end{matrix} \right]\left[ \begin{matrix} V_{i,\to} \\ V_{i,\uparrow} \\ \begin{matrix} V_{i,\leftarrow} \\ V_{i,\downarrow} \end{matrix} \end{matrix} \right]$ (12)

The new basis can be expressed as $V_{i,(\to,\uparrow,\leftarrow,\downarrow)}$=$F{[V}_{i,1},V_{i,2},V_{i,3},V_{i,4}]^{T}$, which construct four decoupled subspaces. Two frequency-dependent terms $V_{i,(\uparrow,\downarrow)}$ acting as a pair of pseudospins $V_{i,\uparrow}=V_{i,1}+V_{i,2}e^{i\pi/2}+V_{i,3}e^{i\pi}+V_{i,4}e^{-i\pi/2}$ and $V_{i,\downarrow}=V_{i,1}+V_{i,2}e^{-i\pi/2}+V_{i,3}e^{i\pi}+V_{i,4}e^{i\pi/2}$. The eigen-equation of the pseudospins $V_{i,\uparrow}$ can be expressed as:

$$\frac{d}{dt}V_{i,\uparrow}=\left( j\frac{2}{CR_{w}}V_{i,\uparrow}+\frac{1}{CR_{3}}-\frac{R_{4}+R_{5}}{100CR_{3}R_{4}}\left| V_{i,\uparrow} \right|^{2} \right)V_{i,\uparrow}-j[\left( \frac{1}{CR_{1}}-\frac{1}{CR_{2}} \right)V_{i+1,\uparrow}+\left( \frac{1}{CR_{1}}+\frac{1}{CR_{2}} \right)V_{i-1,\uparrow}]$$

$+\left( \frac{1}{CR_{1}}-\frac{1}{CR_{2}}-\frac{1}{CR_{1}}-\frac{1}{CR_{2}}+\frac{1}{CR_{a}} \right)V_{i,\uparrow}$*,* (13)

where the circuit parameters are set to ensure $\frac{1}{CR_{1}}-\frac{1}{CR_{2}}-\frac{1}{CR_{1}}-\frac{1}{CR_{2}}+\frac{1}{CR_{a}}=0$. We note that the Supplementary Eq. (13) possesses the same form with Eq. (1) in the main text, where the effective parameters in such designed electric circuit can be expressed as $\omega_{0}=\frac{2}{CR_{w}}$ ,$\alpha=\frac{1}{CR_{3}}$,$\beta=\frac{R_{4}+R_{5}}{100CR_{3}R_{4}}$ , $J_{+}=\frac{1}{CR_{1}}-\frac{1}{CR_{2}}$ and $J_{-}=\frac{1}{CR_{1}}+\frac{1}{CR_{2}}$.

Then, we turn to the nonlinear electric circuits sustaining the non-Hermitian topological global ﻿synchronization. The voltages at four circuit nodes, which work as a single ‘$\alpha$’ or ‘$b$’ sublattice in the *i*th unit, should be written as $V_{i,\alpha}={[V}_{i,\alpha,1},V_{i,\alpha,2},V_{i,\alpha,3}{,V_{i,\alpha,4}]}^{T}$ or $V_{i,b}={[V}_{i,b,1},V_{i,b,2},V_{i,b,3}{,V_{i,b,4}]}^{T}$. Carrying out ﻿Kirchhoff’s law on four circuit nodes (there is no external source) related to *a*-type and *b*-type sublattices, we obtain the following equations as

$$-C\frac{d}{dt}\left[ \begin{matrix} \begin{matrix} V_{i,a,1} \\ V_{i,a,2} \end{matrix} \\ V_{i,a,3} \\ V_{i,a,4} \end{matrix} \right]{=({-R}_{1}}^{-1}+{R_{2}}^{-1})\left[ \begin{matrix} V_{i,a,1}-V_{i,b,2} \\ V_{i,a,2}-V_{i,b,3} \\ \begin{matrix} V_{i,a,3}-V_{i,b,4} \\ V_{i,a,4}-V_{i,b,1} \end{matrix} \end{matrix} \right]+(-R^{-1})\left[ \begin{matrix} V_{i,a,1}-V_{i-1,b,2} \\ V_{i,a,2}-V_{i-1,b,3} \\ \begin{matrix} V_{i,a,3}-V_{i-1,b,4} \\ V_{i,a,4}-V_{i-1,b,1} \end{matrix} \end{matrix} \right]+(R_{a})^{-1}\boldsymbol{I}_{4\times4}\left[ \begin{matrix} V_{i,a,1} \\ V_{i,a,2} \\ \begin{matrix} V_{i,a,3} \\ V_{i,a,4} \end{matrix} \end{matrix} \right]$$

$+\left[ \begin{matrix} \begin{matrix} 0 & -(R_{w})^{-1} \\ (R_{w})^{-1} & 0 \end{matrix} & \begin{matrix} 0 & (R_{w})^{-1} \\ -(R_{w})^{-1} & 0 \end{matrix} \\ \begin{matrix} 0 & (R_{w})^{-1} \\ -(R_{w})^{-1} & 0 \end{matrix} & \begin{matrix} 0 & -(R_{w})^{-1} \\ (R_{w})^{-1} & 0 \end{matrix} \end{matrix} \right]\left[ \begin{matrix} V_{i,a,1} \\ V_{i,a,2} \\ \begin{matrix} V_{i,a,3} \\ V_{i,a,4} \end{matrix} \end{matrix} \right]+(-\frac{1}{R_{3}}+\frac{R_{4}+R_{5}}{100R_{3}R_{4}}[\left| V_{i,a,1} \right|^{2},\left| V_{i,a,2} \right|^{2},\left| V_{i,a,3} \right|^{2},\left| V_{i,a,4} \right|^{2}])\left[ \begin{matrix} V_{i,a,1} \\ V_{i,a,2} \\ \begin{matrix} V_{i,a,3} \\ V_{i,a,4} \end{matrix} \end{matrix} \right]$ (14)

$$-C\frac{d}{dt}\left[ \begin{matrix} \begin{matrix} V_{i,b,1} \\ V_{i,b,2} \end{matrix} \\ V_{i,b,3} \\ V_{i,b,4} \end{matrix} \right]{=(R_{1}}^{-1}+{R_{2}}^{-1})\left[ \begin{matrix} V_{i,b,1}-V_{i,a,4} \\ V_{i,b,2}-V_{i,a,1} \\ \begin{matrix} V_{i,b,3}-V_{i,a,3} \\ V_{i,b,4}-V_{i,a,2} \end{matrix} \end{matrix} \right]+(R^{-1})\left[ \begin{matrix} V_{i,b,1}-V_{i+1,a,4} \\ V_{i,b,2}-V_{i+1,a,1} \\ \begin{matrix} V_{i,b,3}-V_{i+1,a,2} \\ V_{i,b,4}-V_{i+1,a,3} \end{matrix} \end{matrix} \right]+(-R_{b})^{-1}\boldsymbol{I}_{4\times4}\left[ \begin{matrix} V_{i,b,1} \\ V_{i,b,2} \\ \begin{matrix} V_{i,b,3} \\ V_{i,b,4} \end{matrix} \end{matrix} \right]$$

$+\left[ \begin{matrix} \begin{matrix} 0 & -(R_{w})^{-1} \\ (R_{w})^{-1} & 0 \end{matrix} & \begin{matrix} 0 & (R_{w})^{-1} \\ -(R_{w})^{-1} & 0 \end{matrix} \\ \begin{matrix} 0 & (R_{w})^{-1} \\ -(R_{w})^{-1} & 0 \end{matrix} & \begin{matrix} 0 & -(R_{w})^{-1} \\ (R_{w})^{-1} & 0 \end{matrix} \end{matrix} \right]\left[ \begin{matrix} V_{i,b,1} \\ V_{i,b,2} \\ \begin{matrix} V_{i,b,3} \\ V_{i,b,4} \end{matrix} \end{matrix} \right]+(-\frac{1}{R_{3}}+\frac{R_{4}+R_{5}}{100R_{3}R_{4}}[\left| V_{i,b,1} \right|^{2},\left| V_{i,b,2} \right|^{2},\left| V_{i,b,3} \right|^{2},\left| V_{i,b,4} \right|^{2}])\left[ \begin{matrix} V_{i,b,1} \\ V_{i,b,2} \\ \begin{matrix} V_{i,b,3} \\ V_{i,b,4} \end{matrix} \end{matrix} \right]$ (15)

Performing the diagonalization of Supplementary Eq. (14) and Eq. (15) with a unitary transformation

$F=\frac{1}{\sqrt{4}}\left[ \begin{matrix} \begin{matrix} 1 & 1 \\ 1 & e^{i2\pi/4} \end{matrix} & \begin{matrix} 1 & 1 \\ e^{i4\pi/4} & e^{i6\pi/4} \end{matrix} \\ \begin{matrix} 1 & e^{i4\pi/4} \\ 1 & e^{i6\pi/4} \end{matrix} & \begin{matrix} e^{i8\pi/4} & e^{i12\pi/4} \\ e^{i12\pi/4} & e^{i18\pi/4} \end{matrix} \end{matrix} \right]$, (16)

we have

$$-C\frac{d}{dt}\left[ \begin{matrix} \begin{matrix} V_{i,a,\to} \\ V_{i,a,\uparrow} \end{matrix} \\ V_{i,a,\leftarrow} \\ V_{i,a,\downarrow} \end{matrix} \right]={(R_{2}}^{-1}-{R_{1}}^{-1}-R^{-1}+{R_{a}}^{-1})\left[ \begin{matrix} V_{i,a,\to} \\ V_{i,a,\uparrow} \\ \begin{matrix} V_{i,a,\leftarrow} \\ V_{i,a,\downarrow} \end{matrix} \end{matrix} \right]+(\frac{R_{4}+R_{5}}{100R_{3}R_{4}}[\left| V_{i,a,\to} \right|^{2},\left| V_{i,a,\uparrow} \right|^{2},\left| V_{i,a,\leftarrow} \right|^{2},\left| V_{i,a,\downarrow} \right|^{2}]-\frac{1}{R_{3}})\left[ \begin{matrix} V_{i,a,\to} \\ V_{i,a,\uparrow} \\ \begin{matrix} V_{i,a,\leftarrow} \\ V_{i,a,\downarrow} \end{matrix} \end{matrix} \right]$$

$-\frac{R_{1}-R_{2}}{R_{1}R_{2}}\left[ \begin{matrix} \begin{matrix} 1 & 0 \\ 0 & e^{\frac{i\pi}{2}} \end{matrix} & \begin{matrix} 0 & 0 \\ 0 & 0 \end{matrix} \\ \begin{matrix} 0 & 0 \\ 0 & 0 \end{matrix} & \begin{matrix} e^{i\pi} & 0 \\ 0 & e^{-\frac{i\pi}{2}} \end{matrix} \end{matrix} \right]\left[ \begin{matrix} V_{i,b,\to} \\ V_{i,b,\uparrow} \\ \begin{matrix} V_{i,b,\leftarrow} \\ V_{i,b,\downarrow} \end{matrix} \end{matrix} \right]+\frac{1}{R_{1}}\left[ \begin{matrix} \begin{matrix} 1 & 0 \\ 0 & e^{\frac{i\pi}{2}} \end{matrix} & \begin{matrix} 0 & 0 \\ 0 & 0 \end{matrix} \\ \begin{matrix} 0 & 0 \\ 0 & 0 \end{matrix} & \begin{matrix} e^{i\pi} & 0 \\ 0 & e^{-\frac{i\pi}{2}} \end{matrix} \end{matrix} \right]\left[ \begin{matrix} V_{i-1,b,\to} \\ V_{i-1,b,\uparrow} \\ \begin{matrix} V_{i-1,b,\leftarrow} \\ V_{i-1,b,\downarrow} \end{matrix} \end{matrix} \right]-2i\left[ \begin{matrix} \begin{matrix} 0 & 0 \\ 0 & (R_{w})^{-1} \end{matrix} & \begin{matrix} 0 & 0 \\ 0 & 0 \end{matrix} \\ \begin{matrix} 0 & 0 \\ 0 & 0 \end{matrix} & \begin{matrix} 0 & 0 \\ 0 & -(R_{w})^{-1} \end{matrix} \end{matrix} \right]\left[ \begin{matrix} V_{i,a,\to} \\ V_{i,a,\uparrow} \\ \begin{matrix} V_{i,a,\leftarrow} \\ V_{i,a,\downarrow} \end{matrix} \end{matrix} \right]$ (17)

and

$$-C\frac{d}{dt}\left[ \begin{matrix} \begin{matrix} V_{i,b,\to} \\ V_{i,b,\uparrow} \end{matrix} \\ V_{i,b,\leftarrow} \\ V_{i,b,\downarrow} \end{matrix} \right]={(R_{1}}^{-1}+{R_{2}}^{-1}+R^{-1}-{R_{b}}^{-1})\left[ \begin{matrix} V_{i,b,\to} \\ V_{i,b,\uparrow} \\ \begin{matrix} V_{i,b,\leftarrow} \\ V_{i,b,\downarrow} \end{matrix} \end{matrix} \right]+(\frac{R_{4}+R_{5}}{100R_{3}R_{4}}[\left| V_{i,b,\to} \right|^{2},\left| V_{i,b,\uparrow} \right|^{2},\left| V_{i,b,\leftarrow} \right|^{2},\left| V_{i,b,\downarrow} \right|^{2}]-\frac{1}{R_{3}})\left[ \begin{matrix} V_{i,b,\to} \\ V_{i,b,\uparrow} \\ \begin{matrix} V_{i,b,\leftarrow} \\ V_{i,b,\downarrow} \end{matrix} \end{matrix} \right]$$

$-\frac{R_{1}+R_{2}}{R_{1}R_{2}}\left[ \begin{matrix} \begin{matrix} 1 & 0 \\ 0 & e^{-\frac{i\pi}{2}} \end{matrix} & \begin{matrix} 0 & 0 \\ 0 & 0 \end{matrix} \\ \begin{matrix} 0 & 0 \\ 0 & 0 \end{matrix} & \begin{matrix} e^{i\pi} & 0 \\ 0 & e^{\frac{i\pi}{2}} \end{matrix} \end{matrix} \right]\left[ \begin{matrix} V_{i,a,\to} \\ V_{i,a,\uparrow} \\ \begin{matrix} V_{i,a,\leftarrow} \\ V_{i,a,\downarrow} \end{matrix} \end{matrix} \right]-\frac{1}{R_{1}}\left[ \begin{matrix} \begin{matrix} 1 & 0 \\ 0 & e^{-\frac{i\pi}{2}} \end{matrix} & \begin{matrix} 0 & 0 \\ 0 & 0 \end{matrix} \\ \begin{matrix} 0 & 0 \\ 0 & 0 \end{matrix} & \begin{matrix} e^{i\pi} & 0 \\ 0 & e^{\frac{i\pi}{2}} \end{matrix} \end{matrix} \right]\left[ \begin{matrix} V_{i+1,a,\to} \\ V_{i+1,a,\uparrow} \\ \begin{matrix} V_{i+1,a,\leftarrow} \\ V_{i+1,a,\downarrow} \end{matrix} \end{matrix} \right]-2i\left[ \begin{matrix} \begin{matrix} 0 & 0 \\ 0 & \frac{1}{R_{w}} \end{matrix} & \begin{matrix} 0 & 0 \\ 0 & 0 \end{matrix} \\ \begin{matrix} 0 & 0 \\ 0 & 0 \end{matrix} & \begin{matrix} 0 & 0 \\ 0 & -\frac{1}{R_{w}} \end{matrix} \end{matrix} \right]\left[ \begin{matrix} V_{i,b,\to} \\ V_{i,b,\uparrow} \\ \begin{matrix} V_{i,b,\leftarrow} \\ V_{i,b,\downarrow} \end{matrix} \end{matrix} \right]$ (18)

The new basis can be expressed as $V_{i,a/b,(\to,\uparrow,\leftarrow,\downarrow)}$=$F{[V}_{i,a/b,1},V_{i,a/b,2},V_{i,a/b,3},V_{i,a/b,4}]^{T}$. The eigen-equation of the pseudospins $V_{i,a/b,\uparrow}$ can be expressed as:

$\frac{d}{dt}V_{i,a,\uparrow}=\left( j\frac{2}{CR_{w}}V_{i,a,\uparrow}+\frac{1}{CR_{3}}-\frac{R_{4}+R_{5}}{100CR_{3}R_{4}}\left| V_{i,a,\uparrow} \right|^{2} \right)V_{i,a,\uparrow}-j[\left( \frac{1}{CR_{1}}-\frac{1}{CR_{2}} \right)V_{i,b,\uparrow}+\left( \frac{1}{CR} \right)V_{i-1,b,\uparrow}]$

$+\left( \frac{1}{CR_{1}}-\frac{1}{CR_{2}}+\frac{1}{CR}-\frac{1}{CR_{a}} \right)V_{i,a,\uparrow}$, (19)

$\frac{d}{dt}V_{i,b,\uparrow}=\left( j\frac{2}{CR_{w}}V_{i,b,\uparrow}+\frac{1}{CR_{3}}-\frac{R_{4}+R_{5}}{100{CR}_{3}R_{4}}\left| V_{i,b,\uparrow} \right|^{2} \right)V_{i,b,\uparrow}-j[\left( \frac{1}{CR_{1}}+\frac{1}{CR_{2}} \right)V_{i,a,\uparrow}+\left( \frac{1}{CR} \right)V_{i+1,a,\uparrow}]$

$+\left( -\frac{1}{CR_{1}}-\frac{1}{CR_{2}}-\frac{1}{CR}+\frac{1}{CR_{b}} \right)V_{i,b,\uparrow}$, (20)

which possess the same form with Eq. (2) in the main text. The effective parameters in the designed electric circuit are $\omega_{0}=\frac{2}{CR_{w}}$ ,$\alpha=\frac{1}{CR_{3}}$ , $\beta=\frac{R_{4}+R_{5}}{100CR_{3}R_{4}}$ , $J=\frac{1}{CR}$ , $J_{-}=\frac{1}{CR_{1}}-\frac{1}{CR_{2}}$ , and $J_{+}=\frac{1}{CR_{1}}+\frac{1}{CR_{2}}$ with the requirements of $\frac{1}{CR_{1}}-\frac{1}{CR_{2}}+\frac{1}{CR}-\frac{1}{CR_{a}}=-\frac{1}{CR_{1}}-\frac{1}{CR_{2}}-\frac{1}{CR}+\frac{1}{CR_{b}}=0$.

**Supporting Information 12. Simulation results of non-Hermitian skin ﻿synchronization in electric circuits.** In this part, we perform the numerical simulation on voltage dynamics of non-Hermitian skin ﻿synchronic circuits with different lattice lengths. Here, the related parameters are the same to that used in Fig. 4. Figures S15a-S15c presents simulated voltage dynamics and frequency spectra of all circuit oscillators with N=5, 9, and 11, respectively. The corresponding steady-state spatial profiles at t=297.41ms, 299.85ms and 299.81ms are plotted in Figures S15d-S15f. It is clearly shown that the non-Hermitian skin ﻿synchronization appears in the circuit when the lattice length is long enough, being consistent with experimental results.

**
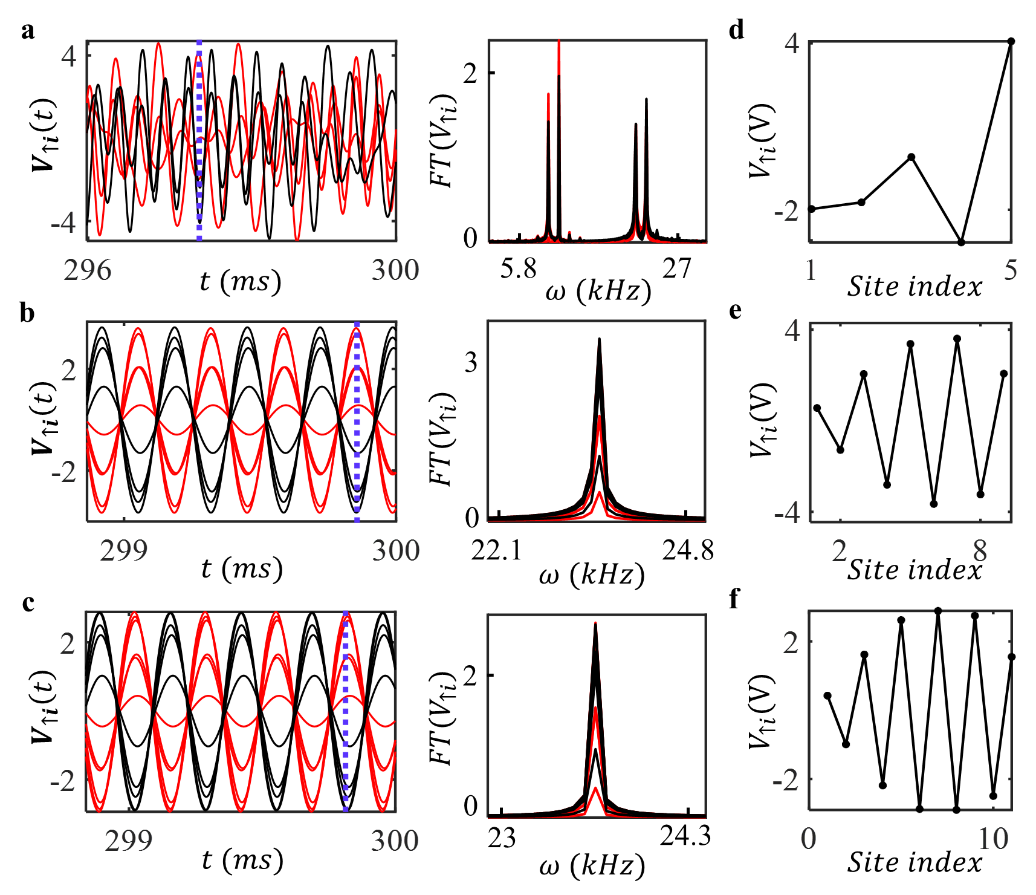
**

**Figure S15. Simulation results of voltage dynamics of the circuit sustaining non-Hermitian skin synchronization with different lattice lengths.** (a)-(c). The voltage dynamics and frequency spectra of the circuit with the lattice length being $N=5, 9, and 11$, respectively. The corresponding spatial profiles at $t=297.41ms,299.85ms,and 299.81ms$ are plotted in (d)-(f).

**Supporting Information 13. Simulation results of non-Hermitian topological global synchronization in electric circuits.** In this part, we simulate voltage dynamics of the non-Hermitian topological global ﻿synchronic circuit with different lattice lengths. Here, the values of related parameters are the same to that used in Fig. 5. Figures S16a-S16c show the voltage dynamics and frequency spectra of all circuit nodes with the lattice length being N=5, 9, and 15, respectively. The corresponding spatial profiles at t=29.96ms, 29.98ms and 29.95ms are plotted in Figures S16d-S16f. It is clearly shown that the non-Hermitian topological global ﻿synchronization appears in the circuit when the lattice length is long enough, being consistent with experimental results.


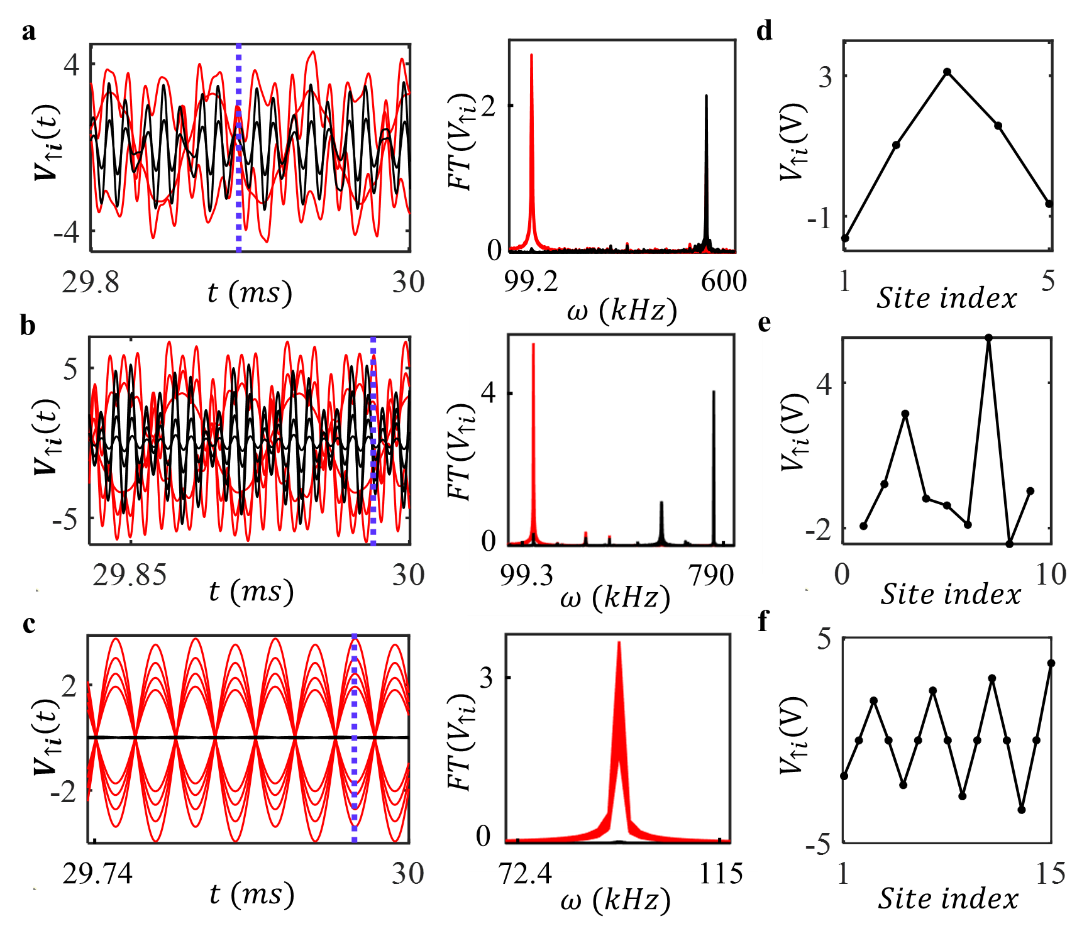


**Figure S16. Simulation results of voltage dynamics of the circuit sustaining non-Hermitian topological global ﻿synchronization with different lattice lengths.** (a)-(c)**.** The voltage dynamics and frequency spectra of the circuit with the lattice length being $N=5, 9, and 15$ respectively. The corresponding spatial profiles at $t=29.96ms,29.98ms,and 29.95ms$ are plotted in (d)-(f)**.**

**Supporting Information 14. Non-Hermitian skin ﻿synchronization in one-dimensional lattices with onsite loss and gain.** In this part, we have added numerical results on non-Hermitian global synchronization based on a non-Hermitian lattice model, where the corresponding non-Hermitian properties are originated from onsite loss and gain. We can see that, except for the specific systems that we considered in the original manuscript, our proposed non-Hermitian global synchronization can also exist in a variety of non-Hermitian Hamiltonians that sustain non-Hermitian skin effects. To illustrate the generalizability of non-Hermitian skin synchronization, we further investigate the nonlinear dynamics based on another representative non-Hermitian lattice model, where the corresponding non-Hermitian properties are purely originated from onsite loss and gain. The schematic diagram of the considered lattice model is plotted in Figure S17a, along with a Stuart-Landau oscillator at each site. Here, each unit contains two sublattices A and B, where the A (red dots) and B (blue dots) sublattices possess onsite gain and loss, denoted by $\pm i\gamma$, respectively. The intracell and intercell couplings between A and B sublattices are $t_{1}$ and $t_{2}$, respectively. The intercell couplings between a pair of A-sublattices or a pair of B-sublattices are $\pm it_{3}$, which can break the time-reversal symmetry of the system. Figure S17b shows spatial profiles of all linear eigenmodes with $t_{1}$=1, $t_{2}=0.6$, $t_{3}=0.2$, *N*=30. It is shown that all eigenstates are localized on the right boundary, manifesting the existence of non-Hermitian skin effect. The system dynamical equations are expressed as

$\dot{Z_{l,A}}=\left( i\omega_{0}+\alpha-\beta| Z_{l,A}|^{2} \right)Z_{l,A}-i\left( {{{t_{2}Z}_{l-1,B}+t}_{1}Z}_{l,B}+{t_{3}e}^{-i0.5\pi}Z_{l-1,A}+{t_{3}e}^{i0.5\pi}Z_{l+1,A} \right)+\gamma Z_{l,A}$ $\dot{Z_{l,B}}=\left( i\omega_{0}+\alpha-\beta| Z_{l,B}|^{2} \right)Z_{l,B}-i\left( {{{t_{2}Z}_{l+1,A}+t}_{1}Z}_{l,A}+{t_{3}e}^{-i0.5\pi}Z_{l-1,B}+{t_{3}e}^{i0.5\pi}Z_{l+1,B} \right)-\gamma Z_{l,B}$. (19)

Numerical results of in-phase and anti-phase synchronized oscillations are shown in Figures S17c-S17d with $\omega_{0}=0.1$, $\alpha=5e^{-3},$ and $\beta=5e^{-2}$. A thousand of random initial states all evolve into one of these two synchronized oscillations. Figures S17e-S17f and Figures S17g-S17h present the corresponding FT frequency spectra and steady-state profiles (at time marked by dash lines). It is shown that the loss and gain-induced non-Hermitian lattice model can also exhibit initial-state-independent global synchronization. Specifically, the spatial profiles and oscillation frequencies of in-phase and anti-phase synchronized states are also precisely matched to eigen-energies and spatial profiles of two linear non-Hermitian skin states, as shown in Figures S17i-S17j, with the normalized factor being 1.73. We also perform the linear stability analysis by linearizing Eq. (19) around anti-phase and in-phase synchronized states, demonstrating that both synchronized states are stable.

Above results clearly shown that non-Hermitian global synchronization can exist in non-Hermitian Hamiltonians with skin effects, where the non-Hermiticity solely arises from onsite loss and gain. It is important to note that on-site loss and gain are well-controlled non-Hermitian terms in both classical and quantum systems, making our proposed non-Hermitian global synchronization become feasible in other practical systems. Therefore, our findings provide a general theoretical framework for constructing non-Hermitian global synchronization, which can be engineered in diverse non-Hermitian systems with non-reciprocal couplings or loss and gain.


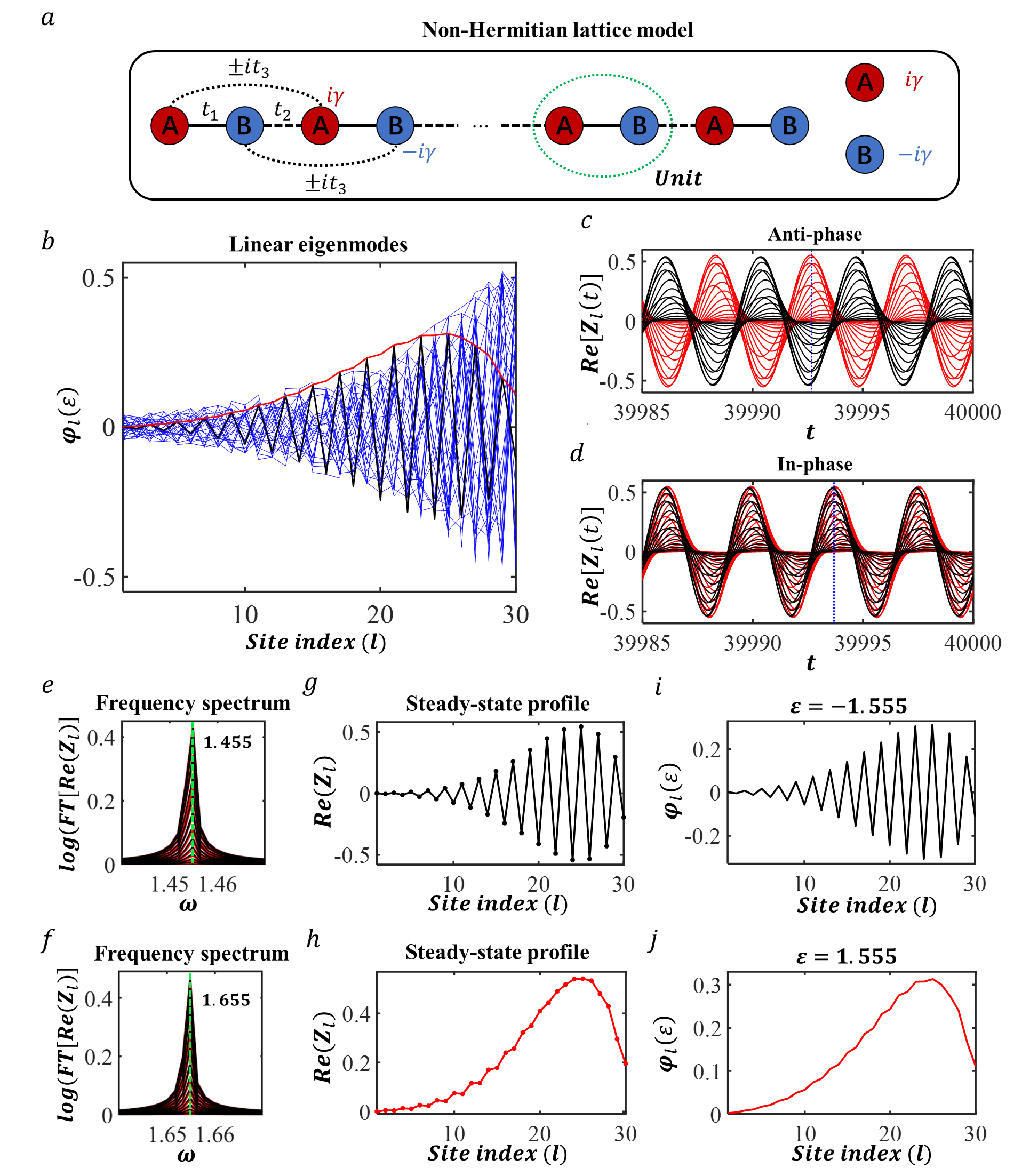


**Figure S17.** (a). The schematic diagram of the non-Hermitian lattice model. (b). Spatial profiles of all linear eigenmodes. (c)-(d). Numerical results of in-phase and anti-phase synchronized oscillations. (e)-(f) and (g)-(h). The corresponding FT frequency spectra and steady-state profiles (at time marked by dashed lines). (i) and (j). Spatial profiles of two linear non-Hermitian skin states. Here, systematic parameters are $t_{1}$=1, $t_{2}=0.6$, $t_{3}=0.2$, N=30, $\omega_{0}=0.1$, $\alpha=5e^{-3},$ and $\beta=5e^{-2}$.

**Supporting Information 15. Sample fabrications and circuit measurements.** The circuit design is conducted using the LCEDA program software, which involves engineering suitable PCB composition, stack-up layout, internal layer design, and grounding design. The designed PCBs consist of six layers with two power layers (±15V), two grounding layers, and all internal layers connected through blind buried holes. For the implementation of the INIC, we utilize LT1363 operational amplifier (OpAmp) due to its excellent performance in experiments. Additionally, two surface-mounted resistors serve as auxiliary resistors in the positive and negative feedback loops of the OpAmp. Both OpAmps and multipliers (AD633JNZs) are powered by external voltages of ±15V. All capacitors and resistors are packaged in 0603 form factor for compactness. Furthermore, a WK6500B impedance analyzer is employed to select circuit elements with high accuracy (disorder strength only 1%) to ensure tolerance of circuit elements. The values of all circuit elements are sufficiently large to neglect any influence from effective resistances or parasitic capacitances in the circuit sample.

As for the time-domain voltage measurement, four circuit nodes, which act as a single lattice site in the tight-binding model, are needed to be excited with suitably phase differences to excite the circuit pseudospin. It is worth noting that, a pair of pseudospins $V_{\uparrow i}=(V_{0},iV_{0},-V_{0},-iV_{0})$ and $V_{\downarrow i}=(V_{0},-iV_{0},-V_{0},iV_{0})$ are degenerated in the region with the approximation of linear-eigenstate expansion of nonlinear dynamics being effective. In this case, we can set the input signals at four circuit nodes as $(V_{0},0,-V_{0},0)$ to simultaneously excite these two pseudospins, where the transient measurement profiles possess the same behavior with that by uniquely excite one of pseudospins $V_{\uparrow i}=(V_{0},iV_{0},-V_{0},-iV_{0})$ or $V_{\downarrow i}=(V_{0},-iV_{0},-V_{0},iV_{0})$. In this case, to initialize the circuit in experiments, each node should be connected to an external voltage signal of $+V_{0}$ , $-V_{0}$ or 0. We use relay model G6K (Omron) to connect the circuit nodes and the external voltage sources. The relays are controlled by a signal of 5V through a mechanical switch. With this setting, the external signals can be removed at the same time. Thus, we connect the nodes to an oscilloscope by coaxial cables and measure the voltage signal after the switch is turned off. It is noted that we measure the voltage at a fixed node (the first one), where voltages at other three nodes are consistent with that of the first one with the linear expansion being effective. Additionally, a 4-channel oscilloscope DSO7104B (Agilent Technologies) is used in experiments to collect the time-domain voltage signals. For each circuit, we made three rounds of measurements and at least 20 times per round to verify the reproducibility of the obtained results.
